# Accurate pain reporting training diminishes the placebo response: Results from a randomised, double-blind, crossover trial

**DOI:** 10.1371/journal.pone.0197844

**Published:** 2018-05-24

**Authors:** Roi Treister, Oluwadolapo D. Lawal, Jonathan D. Shecter, Nevil Khurana, John Bothmer, Mark Field, Steven E. Harte, Grant H. Kruger, Nathaniel P. Katz

**Affiliations:** 1 Department of Neurology, Massachusetts General Hospital, Harvard Medical School, Boston, Massachusetts, United States of America; 2 Faculty of Social Welfare and Health Sciences, University of Haifa, Haifa, Israel; 3 Analgesic Solutions, Natick, Massachusetts, United States of America; 4 Grunenthal GmbH, Aachen, Germany; 5 Chronic Pain and Fatigue Research Center, Department of Anesthesiology, University of Michigan, Ann Arbor, Michigan, United States of America; 6 Department of Mechanical Engineering, University of Michigan, Ann Arbor, Michigan, United States of America; 7 Department of Anaesthesiology and Perioperative Medicine, Tufts University School of Medicine, Boston, Massachusetts, United States of America; National Taiwan University, school of dentistry, TAIWAN

## Abstract

Analgesic trials frequently fail to demonstrate efficacy of drugs known to be efficacious. Poor pain reporting accuracy is a possible source for this low essay-sensitivity. We report the effects of Accurate-Pain-Reporting-Training (APRT) on the placebo response in a trial of Pregabalin for painful-diabetic-neuropathy. The study was a two-stage randomized, double-blind trial: In Stage-1 (Training) subjects were randomized to APRT or No-Training. The APRT participants received feedback on the accuracy of their pain reports in response to mechanical stimuli, measured by R-square score. In Stage-2 (Evaluation) all subjects entered a placebo-controlled, cross-over trial. Primary (24-h average pain intensity) and secondary (current, 24-h worst, and 24-h walking pain intensity) outcome measures were reported. Fifty-one participants completed the study. APRT patients (*n* = 28) demonstrated significant (*p* = 0.036) increases in R-square scores. The APRT group demonstrated significantly (p = 0.018) lower placebo response (0.29 ± 1.21 vs. 1.48 ± 2.21, mean difference ± SD = -1.19±1.73). No relationships were found between the R-square scores and changes in pain intensity in the treatment arm. In summary, our training successfully increased pain reporting accuracy and resulted in a diminished placebo response. Theoretical and practical implications are discussed.

## Introduction

High rate of failed clinical trials is a concern in trials of neurological and psychiatric indications including mood disorders, [1–3] Alzheimer’s disease, [4,5] and pain. [6,7] A trial is considered as failed when a drug known or strongly suspected to be efficacious, fails to show an effect. Common to these indications is the use of subjective outcome measures which are vulnerable to bias and variance. The negative impact of variance in symptom reporting on trial assay sensitivity (the ability of the trial to discriminate a truly active treatment from placebo) is a well-established concern. Another important factor underlying increasing failure rates of clinical trials is the placebo response. [8,9]

Several studies have demonstrated relations between pain score variability and the placebo response. Harris et al. (2005) demonstrated that high variability in baseline pain ratings predicted a high placebo response.[10] This initial observation was later confirmed in a meta-analysis that identified variability in baseline 7-day pain diary reporting as a factor contributing to high placebo response. [11] These relations are not unique for pain: Instability of depression severity at baseline, assessed as the difference between two pre-treatment evaluations, predicted the placebo response in a clinical trial of major depressive disorder. [12]

In a recent study, [13] we developed a method aimed to assess subjects’ pain reporting accuracy. The method (named FAST, the Focused Analgesia Selection Test) is based on recording subjects’ pain reports in a response to the administration of noxious stimuli of various intensities. Each intensity is applied multiple times, allowing assessment of the correlation coefficient (Pearson’s R^2^) between stimuli intensities and pain intensity reports (i.e. subjects’ pain reports accuracy). In this study [13] a cohort of 88 osteoarthritis of the hip, knee, and/or ankle subjects underwent the FAST, based on application of thermal noxious stimuli, at baseline, and then reported their current clinical pain before and after preforming an exercise (stairs climbing) aimed to induce changes in their clinical pain. Results demonstrated that pain reporting accuracy varied between subjects. In addition, subjects’ performance in the FAST correlated with changes in clinical pain: Subjects who were more accurate in reporting pain in response to the FAST also reported larger increase in clinical pain following exercise, than subjects who were less accurate. These results highlighted the relevance of subjects’ performance in the experimental FAST procedure to their ability to report changes in clinical pain.

The objectives of the current study were to assess if pain reporting accuracy could be improved by training, and if the training will affect the placebo response, and consequently, the trial assay sensitivity. Toward this, we developed the Accurate Pain Reporting Training (APRT) program. The APRT is based on applying the FAST multiple times, while providing feedback on pain reporting accuracy between each FAST application. To assess the effects of the APRT, a two-stage randomized controlled trial was conducted. Patients with painful diabetic neuropathy (PDN) were first randomized into Training and No-Training interventions. Subsequently, trained and untrained patients were randomized into a double-blind crossover trial of Pregabalin (PGN). We hypothesized that APRT would increase subjects’ reporting accuracy and that lower placebo response will be observed in the trained cohort.

## Materials and methods

### Study design

We conducted this two-stage methodological study across four clinical sites in the United States. The Training stage was designed as a randomized parallel un-blinded study and the subsequent Evaluation stage was a randomized placebo-controlled double-blind crossover study. The study was reviewed and approved by Asentral, an independent Institutional Review Board (IRB). The study was registered in ClinicalTrials.gov, registration number NCT02842554. The study originally included another training arm that was terminated soon after study initiation due to methodological challenges. The study protocol, CONSORT Checklist, and per-protocol dataset are part of the supporting information of this paper (S1, S2 and S3 Supporting Information respectively).

### Subjects

Subjects were recruited from the community using IRB-approved local and direct (based on clinical site databases) marketing approaches. All subjects provided written informed consent. Eligible subjects were aged 18 years or over, had a clinical diagnosis of PDN for at least six months, and had a 24-h recall average spontaneous pain intensity scores, score of ≥ 4 on the 0–10 numerical rating scale (NRS) as well as an average spontaneous daily pain intensity ≥ 4 for at least 20 of the previous 30 days. We excluded subjects with any chronic pain syndrome that might have interfered with the self-assessment of PDN symptoms. Other exclusion criteria included pregnancy or lactation, unstable coronary artery disease, stroke, uncontrolled hypertension, allergy or refractory status to PGN or gabapentin, or a history (within last 5-years) of epilepsy or other seizure disorder, congestive heart failure, significant gastrointestinal disease, alcohol or drug abuse, or clinically significant altered sensation over the finger to be tested with mechanical pressure.

### Randomization and masking

Randomization was performed in blocks of four on a per-site basis using a computerized randomization system with envelope-based allocation concealment. Each subject was randomly assigned a two-digit number indicating the training-type in the Training stage (1:1, Training or No-Training) and a treatment-type in the Evaluation stage (1:1, PGN–placebo or placebo–PGN). In the Evaluation stage, all subjects, physicians, and study personnel were blinded to treatment sequence assignments and medication codes. Masking was enforced by the over-encapsulation of PGN and placebo in identical capsules and by the use of identical blister packaging that displayed only the subjects’ number, treatment sequence assignment and dosing instructions. Study personal preforming the second study stage were not blinded to allocation into training condition (training/no training).

### Procedures

**Fig 1:**
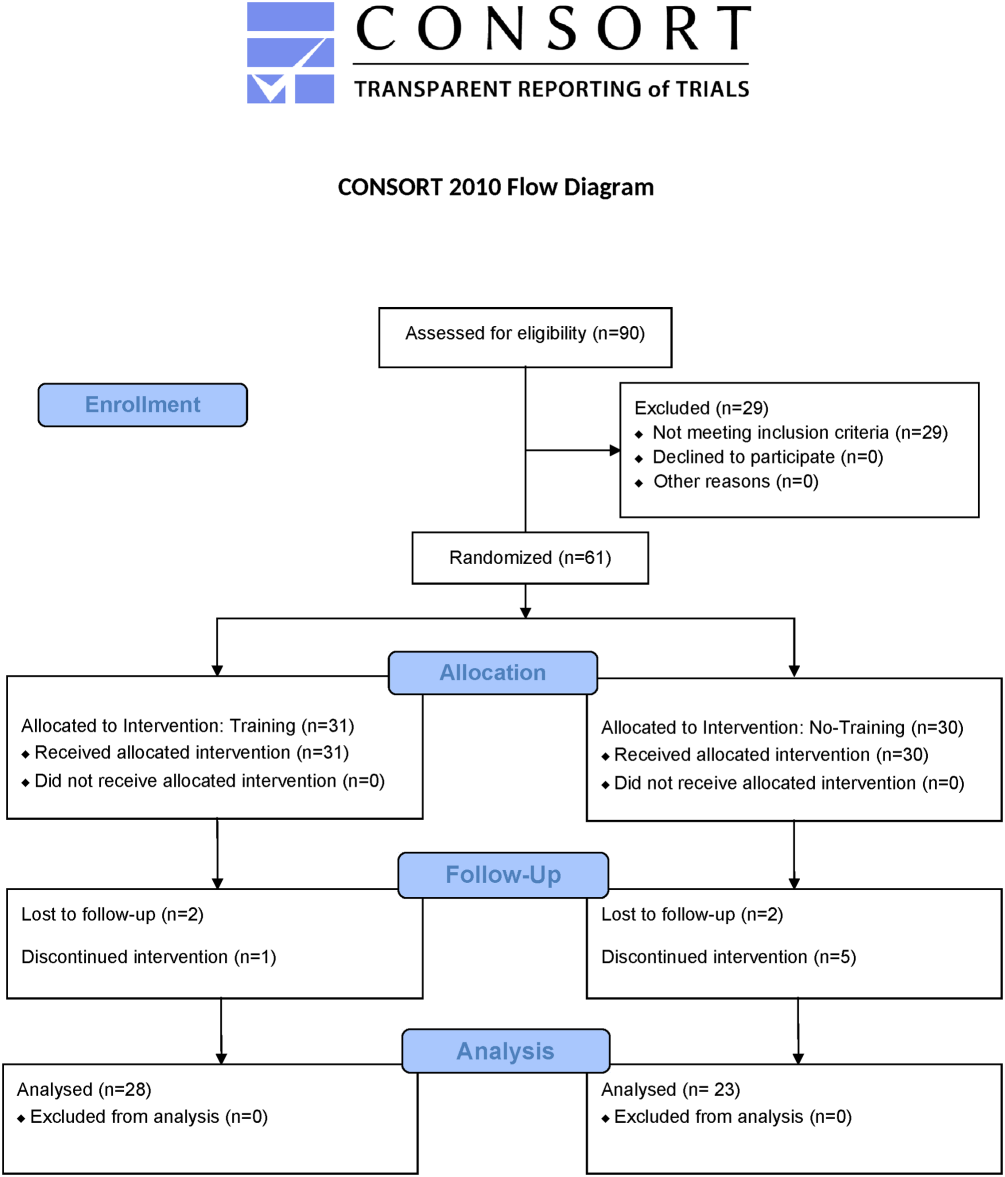
CONSORT participant flow diagram.

**Fig 2:**
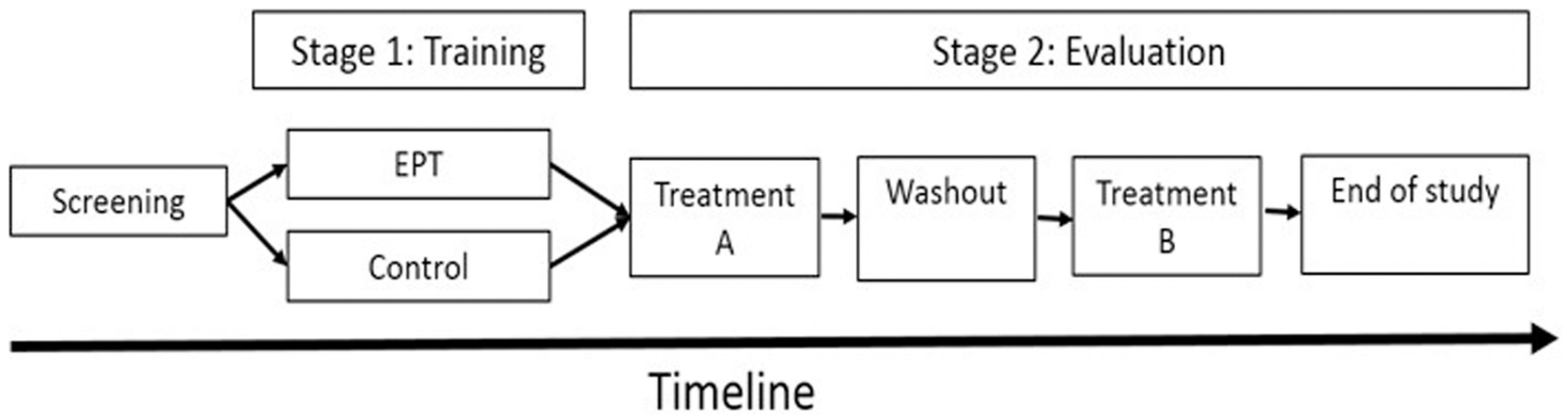
Study design. The study included 2 phases: An unblinded parallel-design training stage, and a double-blind crossover evaluation stage.

The CONSORT participant flow diagram and study design are summarized in Figs 1 and 2 respectively. At the screening visit, baseline demographic information, medical history, and previous treatment experience were recorded and a general physical examination, basic blood chemistry, and pain assessments were conducted. Subjects who met the preliminary entry criteria were enrolled into the Training stage and randomized into the Training arm or the No-Training.

Subjects in the Training arm completed a minimum of two and a maximum of four in-clinic Training sessions (one session every 7±3 days). The original number of sessions was determined to be 4 sessions, but in an attempt to reduce burden and improve retention, the protocol was amended to allow a minimum of two Training sessions. The Multimodal Automated Sensory Testing (MAST) system (University of Michigan, Ann Arbor, MI), was used to apply computer-controlled pressure stimuli to the thumbnail. We chose to base the FAST and the training on pressure stimuli, rather than thermal stimuli as was done in the previous study, to increase the external validity of our approach beyond a specific noxious modality. We assumed that the method will proved useful regardless of stimulus modality, since it is the pain that is assessed by subjects, not the heat or pressure applied. In each Training session, subjects received four discrete applications of six different stimulus intensities, totalling 24 stimuli, each lasting 3 seconds, on each thumbnail bed (a total of 48 stimuli, 24 stimuli per thumbnail). A relatively long Inter-Stimulus-Interval of 30 seconds was applied, to minimize possible peripheral effects (e.g. habituation and sensitisation). At the beginning of each Training session, the 6 stimulus intensities were individually calibrated to range between each subject’s pain threshold (the first pressure rated greater than zero) and 75% of his/her pain tolerance (a point at which a pain rating of 10 was indicated or when the subjects indicated that they could not tolerate a higher pressure). At the end of testing on each thumbnail (i.e. in the halfway of the training procedure and at its end), subjects observed their own rating-versus stimulus intensity scatterplot together with the experimenter. Experimenters were trained to provide standardized feedback (using scripts) focused on the relationship between stimulus intensity and pain scores while directing the subjects’ attention to pain reports that were “outliers”, i.e. pain reports that were far away from other reports of the same stimulus intensity. Experimenters was trained not to suggest that subjects ratings were “wrong”, rather to focus on reinforcing reporting accuracy. Subjects enrolled to the control group received no special training aside from general study procedures.

Upon completion of the Training stage, subjects were randomized into the Evaluation stage. In each treatment period, subjects received PGN or placebo treatment for 10–13 days, 3 times daily, including a titration period of 3–6 days (PGN 150 mg/day) and a stable treatment period of 7 days (PGN 300 mg/day). [14,15] The 24-hour recall average spontaneous pain intensity score served as the primary outcome measure. Current pain intensity, 24-h worst pain intensity, and 24-h average walking pain intensity (secondary outcome measures) were reported at the beginning (pre-treatment) and end of each treatment period using the 0–10 NRS. After the first treatment period and prior to crossover, subjects underwent a washout period of 6 ± 2 days. No rescue medication was provided. To assess durability of training, during the last in-clinic visit (end of the second treatment period), the mechanical FAST version was conducted in both the APRT and the control groups to evaluate pain reporting accuracy at the end of the study (no feedback was provided).

### Outcome measures

The first study stage, the Training stage, was aimed to assess if training could improve pain reporting accuracy. The primary outcome for this objective was the R^2^ value (averaged from two cycles each containing 24 stimulus responses), as an indicator of accuracy of reporting experimental pain. The second study stage, the Evaluation stage was aimed to achieve the second objective of our study: to assess if the training will affect the placebo response (i.e. change in pain during the placebo arm), and consequently, the trial assay sensitivity (the ‘treatment difference’, which is the change in pain in the drug arm minus the change in pain in the placebo arm). The primary outcome endpoint to support this objective was mean change from pre-treatment in 24-h recall average pain intensity on the 0–10 NRS, where 0 indicated no pain and 10 indicated the worst imaginable intensity of pain. Secondary outcomes included mean changes from pre-treatment in current pain intensity, 24-h worst pain intensity, and 24-h average pain during walking. Adverse events, serious adverse events, and concomitant medications were monitored.

### Statistical analysis

Given the exploratory nature of the training program used in this study, the effect size of any difference between training groups was unknown. To calculate the required sample size for discriminating PGN from placebo, a sample size of 67 participants would be sufficient, based on a known moderate effect size of PGN (Cohen’s d ≈ 0.35) [14,15], a two-sided α (0.025), and a power of 0.80. Data was normally distributed, assessed by Kolmogorov-Smirnov test. Independent t-test and chi-square analyses were used to assess differences in demographic and pre-treatment measures between groups. For the analysis of the predefined primary endpoint, p-value below 0.05 was considered significant. For analyses of the 3 secondary endpoints, Bonferroni’s correction was applied, and only p-values below 0.017 were considered significant.

In the Training stage, subject performance across training sessions was assessed by a repeated measures ANOVA on the session average R^2^ value. All other analyses were based on t-tests, either dependent or independent, as appropriate, based on two-sided alpha level. In addition, for each of the pain endpoints, standard effect sizes (SESs) (change in pain during PGN treatment minus change in pain during placebo treatment divided by the within-subject standard deviation [SD]) were used to compare the efficacy of PGN in trained vs. untrained participants. Data are presented as the mean ± SD unless otherwise specified. All analyses were conducted with SAS version 9.4 (SAS Institute, Cary, NC) and SPSS version 19 (SPSS Inc., Chicago, IL).

## Results

Between August 29, 2013 and October 8, 2015, we screened 90 participants and randomised 61 into the first study stage. Fifty-one subjects were included in the Per-Protocol Population (Fig 2). Twenty-eight subjects were allocated to the Training arm, and 23 to the control. Twenty-four subjects were allocated to the PGN–placebo sequence (13 trained and 11 untrained) and 27 (15 trained and 12 untrained) into the placebo–PGN sequence. Twenty-one (41.2%) were male and 30 (58.8%) were female. No significant differences were found in any of the demographic and pre-treatment characteristics between Trained and untrained groups (Table 1).

**Table 1:** Baseline characteristics of the per-protocol population.

	Pregabalin–Placebo (n = 24)	Placebo–Pregabalin (n = 27)	Training (n = 28)	No-Training (n = 23)
Sex				
Male	10 (42%)	11 (41%)	14 (50%)	7 (30.4%)
Female	14 (58%)	16 (59%)	14 (50%)	16 (69.6%)
Age (years)	60 (9.0)	55 (11.0)	57 (11)	58 (9.9)
Ethnic origin				
White	20 (83%)	23 (85%)	23 (82.1%)	20 (87%)
Black	3 (13%)	4 (15%)	4 (14.3%)	3 (13%)
Other	1 (4%)	0 (0%)	1 (3.6)	0 (0%)
Height (inches)	64.8 (5.3)	64.3 (3.9)	64.7 (5.0)	64.4 (4.4)
Weight (kg)	85.6 (20.5)	89.7 (19.1)	87.1 (18.9)	88.6 (21.0)
NRS 24-hour average pain^§^	6.6 (1.7)	7.0 (1.5)	6.6 (1.4)	7.1 (1.8)
NRS current pain^§^	6.1 (2.1)	6.2 (1.6)	6.2 (1.5)	6.1 (2.1)
NRS 24-hour worst pain^§^	7.6 (1.7)	7.3 (2.0)	7.4 (1.7)	7.7 (1.7)
NRS 24-hour walking pain^§^	7.0 (1.8)	7.3 (2.0)	7.2 (1.6)	7.1 (2.1)

Data are n (%) or mean (SD).

^§^ Values obtained at time of screening.

**Fig 3:**
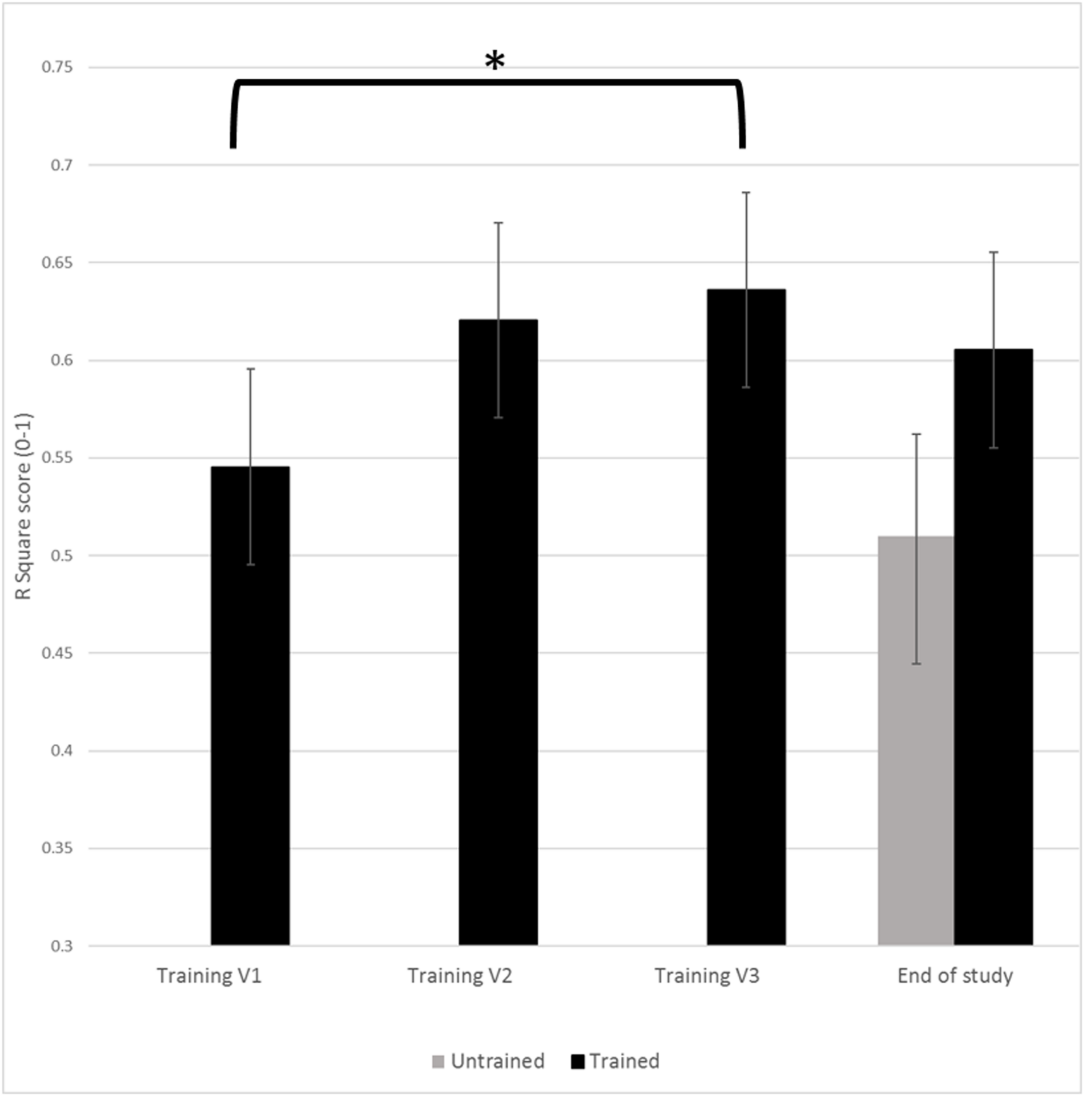
Improved experimental pain reporting accuracy. * = P<0.05.

In the Training stage, the average R^2^ value and number of participants attending each session were: 0.52±0.25 (n = 24) for the first training session, 0.62±0.14 (n = 24) for the second training session, 0.64±0.15 (n = 15) for the third training session, and 0.71±0.17 (n = 3) for the fourth training visit. Given the low number of subjects attending the fourth session, a repeated measures ANOVA including visits 1–3 was conducted and revealed a significant effect of training on R^2^ value (F = 3.75, P = 0.036) (Fig 3), with 70.8% of subjects demonstrated improvement in pain reporting accuracy. Pairwise comparison (dependent t-tests) of the average R^2^ value revealed significant difference between the R^2^ value at the beginning of training (training visit 1) and the R^2^ at the end of training (training visit 3) (P = 0.034; 95% CI = [−0.21, −0.01]; mean difference ± SD = −0.11±0.18), indicating improvements in pain reporting accuracy due to training. No significant difference in R^2^ values was found between training visit 1 and training visit 2 (P = 0.108; 95% CI = [-0.20, 0.02]; mean difference ± SD = -0.09±0.25). At the end of study (i.e. at the end of the second study stage), no significant differences in R^2^ values were seen between trained and untrained participants (P = 0.101; t = 1.68; SES = 0.521).

**Fig 4:**
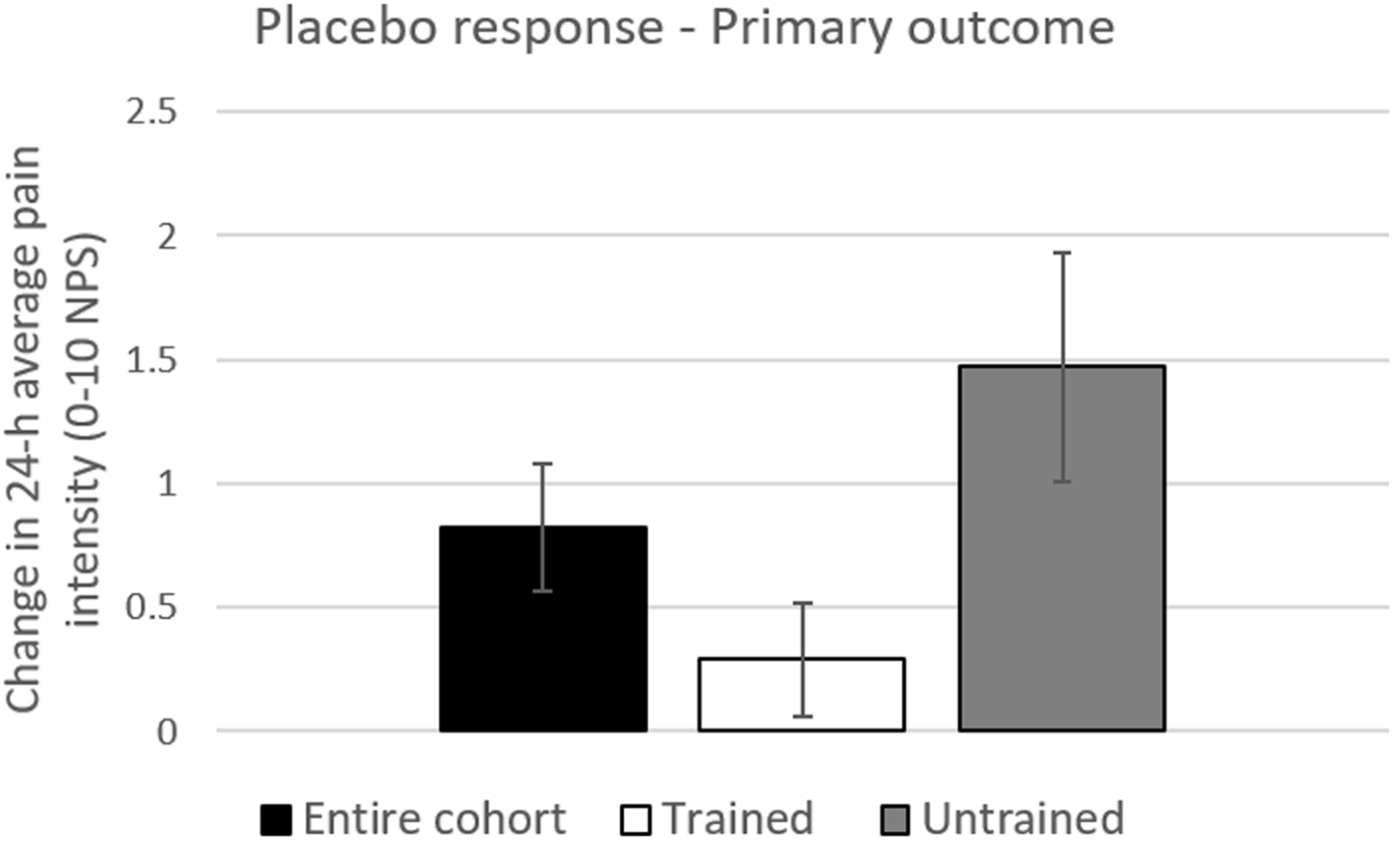
The placebo response in the entire cohort, trained and untrained subjects—Primary outcome measure. Change in placebo was calculated as difference between pain scores in the placebo arm (pre-minus post treatment). Black bars represent changes in pain in the entire cohort. White and Black bars represent changes in pain in the trained (n = 28) and untrained (n = 23) sub-cohorts, respectively. * = P<0.05; Error bars are Standard Error of the Mean (SEM).

**Fig 5:**
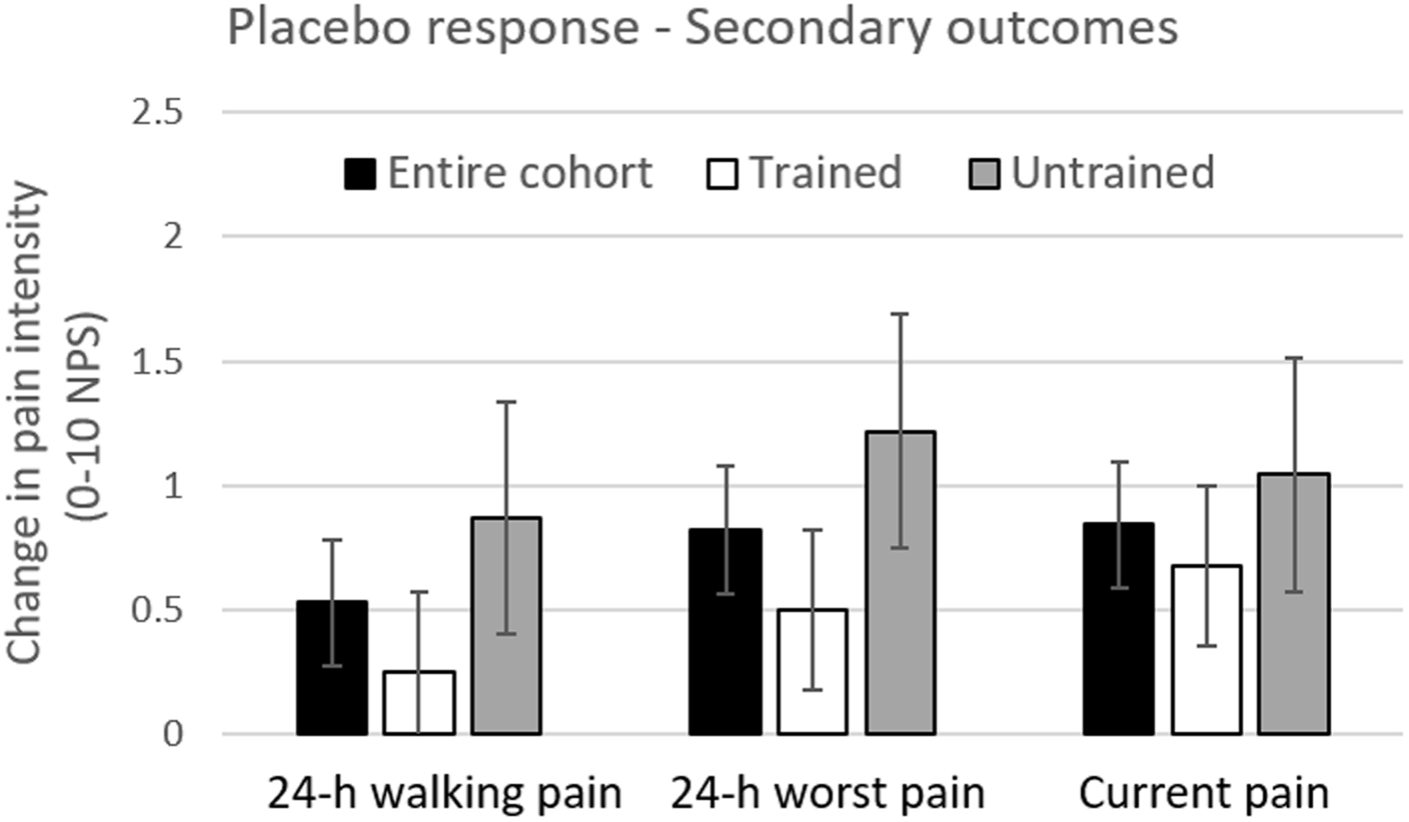
The placebo response in the entire cohort, trained and untrained subjects—Secondary outcome measures. Change in placebo was calculated as difference between pain scores in the placebo arm (pre-minus post treatment). Black bars represent changes in pain in the entire cohort. White and Black bars represent changes in pain in the trained (n = 28) and untrained (n = 23) sub-cohorts, respectively. Error bars are Standard Error of the Mean (SEM).

In the second study stage, for the primary outcome measure, statistically significant difference in the placebo response was found between trained and untrained cohorts (P = 0.018; mean difference ± SD = -1.19±1.73; 95% CI = [-2.17, -0.21]), with smaller placebo responses in trained (0.29±1.21) versus untrained participants (1.48±2.21) (Fig 4). Although no statistically insignificant differences were seen in the placebo response in all secondary outcome measures (following corrections for multiple comparisons), in all measures the placebo responses were numerically smaller in the trained compared to untrained subjects (Fig 5). No statistically significant differences in change in pain in the treatment arm (PGN arm) were found between trained and untrained subgroups in either the primary or secondary measures.

No significant difference between PGN and placebo treatments was found in the primary outcome measure (24-h average pain intensity) (mean treatment difference ± SD = 0.12±2.49; P = 0.73; 95% CI = [-0.58, 0.82]; SES = 0.050). Similarly, no significant differences (following corrections for multiple comparisons) were observed between PGN and placebo treatments in any of the secondary outcome measures): current pain intensity (treatment difference ± SD = 0.08±2.28; P = 0.80; 95% CI = [-0.56, 0.72]; SES = 0.040); 24-h worst pain intensity (treatment difference ± SD = -0.06±2.39; P = 0.861; 95% CI = [-0.73, 0.61]; SES = -0.030); and in the 24-h walking pain, (treatment difference ± SD = 0.73±2.51; P = 0.04; 95% CI = [0.02, 1.43]; SES = 0.290).

When comparing the SES in the trained and untrained subgroups, for the primary measure, the SES was higher in the trained subgroup (SES = 0.31) compared with the untrained subgroup (SES = -0.21). Similarly, SES were higher in the trained subgroup for all three secondary measures: for current pain intensity, SES in the trained subgroup was 0.22, and -0.16 in the untrained subgroup; for 24-h worst pain intensity, SES in the trained subgroup was 0.16, and -0.22 in the untrained subgroup; for 24-h walking pain, SES in the trained subgroup was 0.40 and 0.17 in the untrained subgroup.

## Discussion

We have shown that pain reporting accuracy is a trainable skill that can be improved with training. Training had two effects. First, it improved the accuracy of reporting of experimental pain (i.e., R^2^ value). Second, it reduced the placebo response. If confirmed, the value of improving pain reporting accuracy includes decreased sample size requirements, decreased failed experimentation on human beings, and will accelerate the development of therapeutics by increasing the overall efficiency of clinical development.

The concept of training participants to better report symptoms is relatively novel. In the context of pain research, the Analgesic, Anesthetic, and Addiction Clinical Trial Translations, Innovations, Opportunities, and Networks (ACTTION) initiative attempted to improve trial assay sensitivity by providing participants with training that comprised educational materials on how to more accurately report pain, but the results of this study were inconclusive. [16] Expanding on this premise, we are the first to demonstrate that accuracy of pain reports can be improved by an evoked-pain training approach. At the end of the study, 5 weeks following end of training, no difference was seen between trained and untrained subjects. This suggests that improvement in pain reporting accuracy due to training fades out with time. Hence, re-training might be needed in longer studies.

Our results are consistent with previous studies demonstrating a relationship between variability in pain reporting (presumably driven by error variance) and the placebo response, extending the results of these prior observational studies [10,11] to the interventional context. As in our study, in Harris et al., [10] and in the meta-analysis published later, [11] variability in pain reports associated with changes in pain in the placebo arm, but not with changes in pain in the drug arm, an observation that we currently cannot explain. In these studies, pain reporting variability was calculated based on daily pain scores captured for a 7-day observation period. This variability can be parsed into two components: *true variance* (pain actually does vary day-to-day due to environmental and other influences), plus *error variance* (differences between the patients’ “true” pain intensity and their reported pain intensity). In the training program, our assumption is that we are able to control for (most of) the true variance component by repeatedly applying stimuli of known intensity, such that variability between reports of the same stimuli can mostly be attributed to error variance.

The prevailing consensus is that placebo effect is the result of psychological expectations due to instructions, conditioning, and social learning. [17] Thus, it is unclear why increasing symptom reporting accuracy would impact the placebo response, a phenomenon with sound neurological underpinning [18–20] which is not mere reporting bias. The relationship between the placebo response and variability in pain reports merits future research aimed to increase our understanding of the shared underling mechanisms as well as on its implications for improving assay sensitivity of clinical trials.

In the first FAST study [13] the thermal version of the FAST was used in a cohort of osteoarthritis patients, while in the current study the mechanical FAST (and mechanical APRT) was utilized in a cohort of PDN patients. In both studies the FAST results correlated with changes in clinical pain, thus extending the external validity of this approach beyond stimulus modality and pain indication. In addition, the FAST has been used recently as a test to identify and exclude "poor" pain reporters prior to enrolment in a clinical trial [21,22]

The current study results should be regarded as hypothesis generating, given the pilot nature of this study, the small number of participants and lack of corrections for multiple comparisons. Future research with larger sample size are needed to confirm the results. Two additional limitations deserve consideration: (1) Study staff were not blinded to subjects’ allocation to training/no training. However, given that the second study stage was double blinded, we do not suspect that this affected our results; (2) in the entire cohort, our study failed to discriminate the effects of PGN, a drug known to be efficacious for PDN. These negative results are not entirely unexpected given previous failed PGN trials in PDN populations, and the failures of other efficacious analgesics to demonstrate superiority over placebo in various neuropathic pain indications. [23]

## Conclusions

The results of this study support the hypothesis that training subjects increases pain reporting accuracy, which in turn reduces placebo response. The use of training approaches in future analgesic and potentially other neurological and psychiatric clinical trials has the potential to improve assay sensitivity, reduce sample size requirements, increase the likelihood of trial success, and accelerate the development of new treatment options for those who suffer.

## Supporting information

S1 Supporting Information Asentral Inc IRB approved protocol. (PDF)

S2 Supporting Information CONSORT checklist. (DOC)

S3 Supporting Information Per protocol dataset. (XLSX)
