# Supplementary material for: Accurate pain reporting training diminishes the placebo response: Results from a randomised, double-blind, crossover trial
Source: PLoS One. 2018 May 24;13(5):e0197844. doi: 10.1371/journal.pone.0197844 (PMC5993117; doi:10.1371/journal.pone.0197844)
Supplement: S1 Supporting Information — (PDF) [file pone.0197844.s001.pdf]

CLINICAL STUDY PROTOCOL

**Title:**	**Training "Pain Connoisseurs" for Efficient Analgesic Proof-of-Concept Studies**
**Protocol Number:**	GRN.PC.002
**Study Design:**	Randomized, double-blind, placebo-controlled trial
**Study Phase:**	Methodological Study
**Date:**	August 15, 2013
**Version:**	3.0
**Study Drugs:**	Pregabalin, Oxycodone
**Indication:**	Pain from Diabetic Neuropathy
**Sponsor:**	Analgesic Solutions<br>232 Pond Street<br>Natick, MA 01760
**Sponsor Contacts:**	Nathaniel Katz. MD, MS<br>Analgesic Solutions<br>232 Pond Street<br>Natick, MA 01760<br>Telephone: 781-444-9605 x 242<br>Fax: 508-652-9096<br>E-mail: nkatz@analgesicsolutions.com

The information contained in this document is confidential and is the property of the Sponsor. The information should not be reproduced, revealed, or sent to third parties without the proper authorization and unless agreed to in writing by the Sponsor.

Principal Investigator:

Stephen Wright, MD
Analgesic Solutions
232 Pond Street
Natick, MA 01760
Telephone: 781-444-9605 x 101
Fax: 508-652-9096
E-mail: swright@analgesicsolutions.com

Sub-Investigators:

Nathanial Katz, MD, MS
Analgesic Solutions
232 Pond Street
Natick, MA 01760
Telephone: 781-444-9605 x 124
Fax: 508-652-9099
E-mail: nkatz@analgesicsolutions.com

Protocol Signature Page

The Investigator agrees to conduct the clinical study which is the subject of this protocol in accordance with the Clinical Study Agreement, this protocol, all applicable laws and regulations, and the conditions of approval imposed by the reviewing Institutional Review Board.

Agreed to by the Principal Investigator:

_______Stephen Wright, MD_________________________

Printed Name – Principal Investigator

____

Signature – Principal Investigator

____

Date

Summary of Changes – Version 2.0 to Version 3.0

1. Kelly Wawryzniak has been removed from the study protocol as she is no longer with Analgesic Solutions.
2. Jay Trudeau has been removed from the study protocol as he is no longer with Analgesic Solutions.
3. Nathaniel Katz has been added as the sponsor contact.
4. The end anchor of "10" on the NRS scale was changed from "Extreme Pain" to Worst Pain Imaginable".
5. Randomization numbers were changed.
6. Visit Days were renamed for clarification.
7. Visit 8 was added as an end of study visit.

Asentral, Inc. Institutional Review Board approved – August 22, 2013

TABLE OF CONTENTS

SYNOPSIS **10**
1. INTRODUCTION **20**
1.1. Overview **20**
1.2. Study Design Rationale **21**
1.2.1. General Design Rationale 21
1.2.2. Treatments 22
2. STUDY OBJECTIVES **23**
2.1. Primary Objective **23**
2.2. Secondary Objectives **23**
3. STUDY ENDPOINTS **23**
4. STUDY DESIGN **24**
5. STUDY POPULATION **26**
5.1. Number of Participants **26**
5.2. Duration of Study **26**
5.3. Number of Study Centers **26**
5.4. Eligibility Criteria **26**
5.4.1. Inclusion Criteria 26
5.4.2. Exclusion Criteria 27
5.5. Screening - Visit 1 (Day 1) **34**
5.5.1. Informed Consent 34
5.5.2. Assignment of Subject Number 34
5.5.3. Eligibility 34
5.5.4. Clinical Pain Intensity 35
5.5.5. Focused Analgesia Selection Task (FAST) 35
5.5.6. Training Assignment 35
5.6. Training Stage **36**
5.6.1. Evoked Pain Training Condition - Visits T1 through T4 (Days 2 through 29)36
5.6.1.1. Evoked Pain Training 36

Asentral, Inc. Institutional Review Board approved – August 22, 2013

5.6.2. Drug/Placebo Administration Training – Visits T1 through T4 (Days 2 through 29)....36
5.6.2.1. DPA Treatment Randomization 37
5.6.2.2. Pre-treatment Assessments....37
5.6.2.3. Vital Signs: 37
5.6.2.4. Clinical Pain Intensity: 37
5.6.2.5. DPA Training Stage Treatment 38
5.6.2.6. Post-treatment Assessment and Feedback....38
5.6.3. Control Training Condition 39
5.7. Training Evaluation Stage 39
5.7.1. Visit 2 (Begin Treatment Period A)....39
5.7.1.1. Review of Eligibility Criteria 39
5.7.1.2. Treatment Randomization 39
5.7.1.3. Baseline A Assessments....40
5.7.1.4. Vital Signs 40
5.7.1.5. Pain Intensity 40
5.7.1.6. Dispensing of Study Medication....41
5.7.1.7. Dosing 41
5.7.1.8. Participant Instructions....41
5.7.2. Visit 3 (Tolerability Check)....41
5.7.3. Visit 4 (End of Treatment Period A)....42
5.7.3.1. End of Treatment Period A assessments....42
5.7.3.2. Vital Signs 42
5.7.3.3. Pain Intensity 42
5.7.3.4. PGIC....42
5.7.4. Washout....42
5.7.5. Visit 5 (Begin Treatment Period B) 42
5.7.5.1. Review of Eligibility Criteria 42
5.7.5.2. Baseline B Assessments....43
5.7.5.3. Vital Signs 43
5.7.5.4. Pain Intensity 43
5.7.5.5. Dispensing of Study Medication....43

5.7.5.6. Dosing 43
5.7.5.7. Participant Instructions 44
5.7.6. Visit 6 (Tolerability Check) 44
5.7.7. Visit 7 (End of Treatment Period B, End of Study) 44
5.7.7.1. End of Treatment Period B Assessments 44
5.7.7.2. Vital Signs 44
5.7.7.3. Pain Intensity 45
5.7.7.4. PGIC 45
5.7.7.5. Patient Global Preference for Treatment 45
5.7.7.6. FAST or Psychophysical Profile 45
5.7.8. Withdrawal and/or Early Termination Procedures 45
5.7.9. Unblinding of Subject Treatment 45
6. STATISTICAL METHODS 46
6.1. Study Hypothesis 46
6.2. Study Populations 46
6.3. Sample Size 46
6.4. Randomization and Blinding 47
6.5. Data Analysis 47
6.5.1. General Considerations for Efficacy Analysis 47
6.5.2. Methodology to Achieve Study Objectives 48
6.5.3. Safety Analyses 50
6.6. Interim Analysis 51
6.7. General Statistical Considerations 51
7. ADVERSE EVENTS 51
7.1. Definition of Adverse Events 51
7.2. Definition of Serious Adverse Events 52
7.3. Relationship of Adverse Events to Study Medications 52
7.4. Severity of Adverse Events 53
7.5. Reporting of Adverse Events 53
8. CASE REPORT FORMS 53
9. ADMINISTRATIVE REQUIREMENTS 54
9.1. Investigator Selection 54

Asentral, Inc. Institutional Review Board approved – August 22, 2013

9.2. **Regulatory and Ethical Considerations** **54**
9.3. **Institutional Review Board Approval** **54**
9.4. **Informed Consent** **55**
9.5. **Protocol Adherence and Amendment** **55**
9.6. **Drug Information** **55**
9.6.1. General Information on Pregabalin 55
9.6.2. General information on oxycodone 56
9.6.3. Clinical Supplies 56
9.6.4. Drug Accountability 56
9.6.5. Potential Risks and Discomforts 56
9.6.6. Breaking of the Blind 57
9.6.6.1. During Training Evaluation stage 57
9.6.6.2. During DPA training 57
9.7. **Data Collection** **58**
9.8. **Data Management** **58**
9.9. **Database Quality Assurance** **58**
9.10. **Required Documentation** **58**
10. **SITE MONITORING** **59**
11. **TERMINATION OF STUDY** **59**
12. **REPORTING REQUIREMENTS** **59**
13. **RECORD RETENTION** **59**
13.1. **Financial Disclosure** **60**
13.2. **Use of Information and Disclosure of Data** **60**
14. **REFERENCES** **61**
15. **APPENDICES** **61**
15.1. **Informed Consent Form** .... Error! Bookmark not defined.
15.2. **FAST Assessment** **61**
15.3. **Evoked Pain Training (EPT)** **63**
15.3.1. Assessment of Pain Threshold and Tolerance 63
15.3.2. Index Pain Assessment 64
15.3.3. Random Magnitude Estimation 64
15.3.4. Training feedback 64

15.4. Treatment Experience Questionnaire (TEQ)....66
15.5. Pain Intensity NRS....67
15.6. Patient Global Impression of Change....68
15.7. Patient Preference for Treatment....69
15.8. Participant Training Feedback Questionnaire....70
15.9. BMI Calculation....71
15.10. MAST System Safety Features and Risk Determination....72
15.10.1. MAST System Safety Features....72
15.10.2. MAST Risk Determination....73

LIST of TABLES and FIGURES

Table 1. Screening/Baseline Study Procedures 29
Table 2 Training/Treatment Randomization 40

Figure 1. Study Design....25
Figure 2 EPT 64

SYNOPSIS

Protocol Number: GRN.PC.002

Sponsor: Analgesic Solutions

Title: Training "Pain Connoisseurs" for Efficient Analgesic Proof-of-Concept Studies

Study drug and dose:

Training Stage (Drug/Placebo Administration [DPA] group only):

1) Oxycodone immediate-release (IR) 10mg, single-dose oral
2) Pregabalin 150mg, single-dose oral
3) Matched placebo

Training Evaluation Stage:

1) Pregabalin 150-300mg/day (tid dosing)
2) Matched placebo (tid dosing)

Indication: Relief of pain from painful diabetic neuropathy (PDN)

Study phase: Investigator-initiated methodological study

Number of study centers: 1 (Analgesic Solutions, Natick, MA)

Study objectives:

Primary objective

To assess the ability of Evoked Pain Training (EPT) and Drug/ Placebo Administration (DPA) training to increase subjects' ability to discriminate between active and placebo treatments in a double-blind crossover trial of a known analgesic, measured by standardized effect size, relative to untrained control subjects.

Secondary objective:

To evaluate whether baseline characteristics of subjects predict response to training, measured by differences in psychophysical profile between baseline and end of study.

Study Design:

Participants will be enrolled and evaluated on inclusion/exclusion criteria, including urine pregnancy screen and creatinine clearance, prior to randomization. Participants will be evaluated on baseline characteristics using the Focused Analgesia Selection Task (FAST). The FAST includes completion of a battery of psychosocial assessments (e.g., depression,

anxiety, fear of pain, etc.) and a psychophysical profiling assessment in which participants repeatedly rate a random sequence of painful stimuli[a] and are quantified based on the consistency of their responses.

Training Stage

Qualified participants will be randomized to one of three training conditions for the Training Stage of the study. The Control (C) group will receive no training during the Training Stage. They will roll over directly into the Training Evaluation stage of the study.

The EPT group will receive 4 weekly in-clinic training sessions using repeated rating of painful pressure stimuli applied to the thumbnail via a Multimodal Automated Sensory Testing (MAST) system. Participants undergoing EPT will receive feedback during their training and be evaluated on their pain reporting ability at each session. After 4 training sessions EPT participants will roll over into the Training Evaluation stage. Neither EPT group nor the C group will receive any study medication during the Training Stage.

The Drug/Placebo Administration (DPA) training group will receive 4 weekly in-clinic training sessions using single-dose treatments of active drug or placebo in a 4-period crossover design, 1 treatment per week. Across the 4 sessions participants will receive a single dose of 10mg oxycodone IR, a single dose of 150mg pregabalin, or a single dose of placebo (administered in two periods) in double-blind randomized order. At each session participants will receive a single dose of study medication and stay in the clinic for 4 hours of observation during which they will be evaluated for on their PDN pain every 2 hours. At the end of the session they will be asked if they believe they received an active drug or placebo, be unblinded to the treatment they just received, and receive any additional feedback. After completing the 4-week training program, DPA-trained participants will roll over into the Training Evaluation stage.

Training Evaluation Stage

All participants completing a training session (or no training for the C group) will roll over for randomization into the Training Evaluation stage, which is a randomized double-blind crossover trial of pregabalin vs. placebo. Randomization for the Training Evaluation stage will occur approximately 1 week after completion of training. Participants will be randomized to pregabalin or placebo and enter a 3 day titration period followed by 7 days of stable treatment. Participants having difficulty with titration may be allowed an additional 3 days of titration before the 7-day stable dose period. Treatment Period A ends with in-clinic evaluation and a 7 +/-1 day washout period prior to Treatment Period B. Treatment Period B is an identical 3 day titration (with optional extra 3 days), 7 days of stable treatment, and a final in-clinic assessment. Participants will be assessed at each

[a] Previous versions of the FAST used thermal stimuli, but in order to be consistent with the training paradigms used in this study the FAST will be administered using the same MAST system technology used in the EPT arm.

Asentral, Inc. Institutional Review Board approved – August 22, 2013

clinic visit on a 0-10 Numerical Rating Scale (NRS) for pain intensity average in the past 24 hours, worst pain intensity in the past 24 hours, pain during walking in the past 24 hours, and current pain intensity, as well as a Patient Global Impression of Change (PGIC), and, after Treatment Period B only, Patient Preference for Treatment and FAST.

Number of Participants:

Up to 105 participants will be randomized to the training conditions, intending 30 completers per arm with up to 5 replacements allowed for dropouts from each training condition.

Participants who discontinue early after being randomized to treatment in the Training Evaluation stage will not be replaced. Only 5 replacements will be made in each training condition arm; further dropouts beyond 5 in a training condition will not be replaced.

Eligibility Criteria:

Inclusion criteria:

A subject must meet all of the following criteria to be enrolled in the study:

1) Be a man or a non-pregnant, non-lactating woman 18 years or older. Women of childbearing potential should be willing to use an acceptable birth control method (at the Investigator's discretion) during the study to avoid pregnancy.
2) Have voluntarily provided written informed consent (see attached ICF).
3) Be able to speak, read, write, and understand English, understand the consent form, complete study-related procedures, and communicate with the study staff.
4) Have a clinical diagnosis of Painful Diabetic Neuropathy (PDN) for at least 6 months.
   a. Clinical diagnosis may be verified by medical records or by clinical examination during the first visit combined with a medical history of appropriate symptoms for at least 6 months.
5) Have a pain intensity score averaging ≥4 on a 0-10 NRS for average daily recall over past 24 hours. (This applies at V1, V2, and V5.)
6) Have an average daily pain intensity of at least 4 on the 0-10 NRS on at least 20 out of the past 30 days.
7) Be, in the opinion of the Investigator, in sufficiently good health to participate in the study at screening, based upon the results of a medical history, physical examination and laboratory analysis.

Prior to each Treatment Period, the participants must meet the following additional criteria for randomization: Have average pain intensity (24-hour recall) ≥4 on the 0-10 NRS.

Exclusion criteria:

A subject must be excluded if any of the following criteria are met:

Asentral, Inc. Institutional Review Board approved – August 22, 2013

1. Are pregnant and/or lactating.
2. Have been diagnosed as having any inflammatory arthritis, gout, pseudo-gout, Paget's disease, fibromyalgia or any chronic pain syndrome that in the Investigator's opinion would interfere with the assessment or self-evaluation of pain and other symptoms of PDN.
3. Have evidence for multiple causes of pain in the neuropathic pain area, such as lumbar radiculopathy.
4. Have received or used any of the excluded/prohibited treatments or drugs specified in the list of prohibited treatments (below) or are unable to agree to the list of treatments prohibited during the study.
5. Have a history of congestive heart failure, unstable coronary artery disease, stroke, or uncontrolled hypertension.
6. Have a history of significant gastrointestinal disease, including active gastro-duodenal ulcerations, perforations, or bleeds.
7. Have abnormal clinical laboratory test results or vital signs unless deemed not clinically significant by the investigator.
8. Have regularly worn false fingernails within the past 6 months (more than 25% of the time)
9. Are undergoing active treatment for cancer, are known to be infected by human immunodeficiency virus, or are being acutely and intensively immunosuppressed following transplantation.
10. Have a history of alcohol or other substance abuse (not including nicotine or tobacco) within 5 years.
11. Have a history of suicide attempt within the past 1 year or suicidal ideation within the past 1 month.
12. Have a history of epilepsy or other seizure disorder.
13. Have creatinine clearance below 60 mL/min as calculated by Cockroft-Gault equation for serum creatinine.
14. Known to have a condition that in the Investigator's judgment precludes participation in the study.
15. Have previously been admitted to this study.
16. Are involved in a worker's compensation, disability claim, or litigation related to medical condition or treatment that is open or was settled within the past 12 months. (Whether litigation is related to medical condition or treatment may be decided at the Investigator's discretion. Claims settled >12 months previously are permitted.)
17. Have a known failure to respond to pregabalin, gabapentin, or oxycodone due to either efficacy or tolerability in previous treatment at therapeutically appropriate doses.
18. Are allergic to or have a hypersensitivity to pregabalin or oxycodone.

Prohibited treatments:

Asentral, Inc. Institutional Review Board approved – August 22, 2013

- Use of pregabalin or gabapentin within 2 weeks of the study and for the duration.
- Use of opioids in doses greater than 80mg morphine equivalent per day, either PRN or stable daily doses, within 1 week of study and for the duration of the study.
- Use of prescription capsaicin 8% patch (e.g., Qutenza®) within 3 months. (Over-the-counter topical capsaicin creams [0.025% and 0.075%] are not excluded.)
- Nerve block or intrathecal analgesia within 6 weeks of study.
- Neuro-ablation or neurosurgical intervention for their PDN.
- Any investigational drug or have used an investigational device in the 30 days prior to study entry.
- As-needed (PRN) use of NSAID or opioid compounds (oral or topical) for the duration of the Training Evaluation stage of the study. NSAID or opioids taken at a stable dose and consistent daily regimen are permitted.

The following concomitant medication rules apply specifically to participants in the EPT and DPA training conditions:

Participants in the EPT or DPA training are permitted the use of PRN analgesic medications during the Training stage of the study, provided that they agree not to take any PRN medication on the day of a training visit prior to the visit. PRN analgesic use after completion of a training visit is permitted.

The following concomitant medications are specifically allowed:

- Concomitant medications not specifically excluded are permitted, including analgesics, if they are at stable doses taken on a regular schedule at the Investigator's discretion.
- Use of sliding scale insulin is specifically permitted during the study.

Treatment:

During the Training Stage study medication will be administered only to participants randomized to the DPA group. In the Training stage, DPA participants will receive 1 dose of study medication at the beginning of each of 4 weekly training sessions (treatments allocated in randomized order: 1 pregabalin, 1 oxycodone, and 2 placebo). In the Training Evaluation stage, all participants will receive 10 to 13 days of treatment with either placebo or pregabalin.

Pregabalin (Lyrica®): For DPA Training stage: One 150-mg capsule to be dosed orally 1 time at the beginning of the DPA training session. For Training Evaluation stage: 50-mg capsules to be dosed orally as 1 capsule tid for 3 or 6 days (i.e., 150mg/day) and then 2 capsules tid for 7 days (i.e., 300mg/day).

Asentral, Inc. Institutional Review Board approved – August 22, 2013

Oxycodone: For DPA Training stage: One 10-mg capsule to be dosed orally 1 time at the beginning of DPA training session.

Placebo: Placebo does not contain pregabalin or oxycodone. During the Training stage, matching capsules will be dosed orally 1 time. During the Training Evaluation stage, 1 matching capsule tid for 3 or 6 days and 2 capsules tid for 7 days.

All treatments for the DPA Training stage will be over-encapsulated to be visually indistinguishable for each other. All treatments for the Training Evaluation stage will be over-encapsulated to be visually indistinguishable from each other. DPA treatments may or may not be distinguishable from Training Evaluation stage treatments.

Rescue therapy, dose, and mode of administration:
No rescue medication will be provided for this short-duration study. However certain stable dose concomitant medications with regular dosing schedules are permitted per the protocol.

Duration of study:

Participants will be in the study for up to a maximum of 62 days (68 days with optional extended titration), a period that includes an approximately 4-week training period, 10-day treatment (Treatment Period A), a 7-day washout period, and a 10-day treatment (Treatment Period B). The study will enroll patients for up to 18 months.

Criteria for evaluation:

Primary endpoint 1 (Treatment Efficacy): Overall difference between treatment periods (pregabalin vs. placebo in Training Evaluation stage) in change from period baseline to period end of treatment on 0-10 NRS 24h average pain intensity.

Primary endpoint 2 (Training Efficacy): Difference between training conditions (EPT, DPA training, and C) in the magnitude of treatment effect of pregabalin versus placebo, as measured by change from baseline in 0-10 NRS 24h average pain intensity. I.e., interaction of treatment effect (pregabalin vs. placebo) with training conditions (EPT, DPA, C).

Safety endpoint: Adverse Events (AEs).

Secondary endpoints:

- Change from baseline 0-10 NRS current, 24-hour worst and walking pain intensity
- Patient Global Impression of Change (PGIC)

- Patient Preference for Treatment
- Change from baseline in psychophysical function variables
- Pressure pain threshold and tolerance
- Perceptual power function intercept and slope exponent
- Evoked pain report reliability (coefficient of variation [CoV])

NRS secondary endpoints will be analyzed for treatment efficacy as described above. PGIC will be analyzed similarly but using score at end of treatment rather than change score. Change from baseline to end of study in psychophysical function variables and CoV will be compared by pairwise comparison of training condition groups for training efficacy.

Statistical Methods:

Study hypothesis: The hypotheses in this study are:

(a) Pregabalin will provide more pain relief (a greater difference between baseline and end of treatment) on the primary endpoint than placebo.

(b) The effect size of difference between placebo and pregabalin on the primary endpoint will be greater for participants in the EPT condition than those in the C condition.

(c) The effect size of difference between placebo and pregabalin on the primary endpoint will be greater for participants in the DPA condition than those in the C condition.

(d) The effect size of difference between placebo and pregabalin on the primary endpoint will not differ between those participants in the EPT condition and those in the DPA condition.

Additionally, given the exploratory nature of the study we anticipate evaluating without formal hypotheses which, if any, baseline participant characteristics are predictive of change in pain reporting characteristics (from psychophysical profile) for each training group.

Study populations:

The following populations are defined:

Safety Population: This population will include all participants who receive at least 1 dose of study drug (placebo or pregabalin).

Per-protocol Population: The per-protocol population will include all participants who complete the 2 treatment periods without major protocol violation.

Intent-to-Treat (ITT) Population: The intent-to-treat population will include all participants who receive at least 1 dose of study drug in both treatment periods.

Sample size:

Asentral, Inc. Institutional Review Board approved – August 22, 2013

A total of 105 participants are planned, targeting 90 participants in the Training Evaluation stage and allowing for replacement of up to 5 participants per training arm (15 total) to accommodate training attrition, bringing the total N to up to 105 participants.

Given the exploratory nature of the training programs, the effect size of any difference between groups is unknown. However, the interaction term of an analysis of variance (ANOVA) for 3 conditions by 2 treatments will provide a test of whether training group interacted with the difference between conditions. If we assume testing for marginal significance in this exploratory work (alpha=.10) and a Cohen's *f* of .15 (a medium effect for this measure) a sample of N=90 in 3 groups with 2 measurements provides a power of approximately .80.

Ninety participants also provides sufficient power for the entire sample (across training conditions) to differentiate between placebo and pregabalin. Assuming a moderate effect size of pregabalin in PDN of .35[b] a paired-comparison t-test between means (placebo vs. pregabalin) with alpha=.05 and power=.90 power analysis yields a sample size requirement of N=88. A sample of N=90 allows for convenient balancing of assigned conditions and treatment orders to six cells (3 training conditions and 2 treatment orders).

Data analyses:

The primary efficacy endpoint, difference between treatment periods (pregabalin vs. placebo in Training Evaluation stage) in change from baseline 0-10 NRS 24h average pain intensity will be analyzed by a paired-sample comparison t-test. Mean between-group difference in pain intensity change from baseline for 24-hour worst and average, PGIC, and allodynia will be secondary outcome measures similarly analyzed by paired-sample comparison tests.

Efficacy of the training conditions will be evaluated by comparison of standardized effect size (SES) between conditions and analysis of variance (ANOVA) test of the interaction between treatment condition and training condition.

The ability of baseline characteristics to predict change in pain reporting ability (as measured by psychophysical function) will be modeled by multiple linear regression. Additional models will be constructed for the EPT and DPA groups that will include characteristics specific to each training condition (e.g. ability to discriminate between analgesic and placebo in DPA training).

Safety analyses will include vital signs and AEs during the Training Evaluation stage

[b] This is slightly lower than some published studies to allow a margin for the short treatment period.

and during the single-dose training session for subjects in the DPA arm. AEs will be displayed by body system and preferred term, and analyzed in terms of severity and relationship to study drug. Vital signs analysis will include the mean, standard deviation, minimum, maximum, and quartiles at each visit, as well as Change from Baseline.

All analyses will be conducted with either SAS v9.2 or later or SPSS v19 or later statistical software. No imputation of missing data is planned; subjects will be excluded from any analyses requiring data they are missing.

Interim analysis:

No interim analyses are planned.

Asentral, Inc. Institutional Review Board approved – August 22, 2013

Protocol Abbreviations/Acronyms

Abbreviation/Acronym	Definition
AE	Adverse Event
ALT	Alanine aminotransferase
ANOVA	Analysis of variance
AST	Aspartate aminotransferase
CFR	Code of Federal Regulations
CoV	Coefficient of Variation
CRF	Case Report Form
DPA	Drug/Placebo Administration
DPN	Diabetic Peripheral Neuropathy
EPT	Evoked Pain Training
FAST	Focused Analgesia Selection Task
FDA	U.S. Food and Drug Administration
HADS	Hospital Anxiety and Depression Scale
ICC	Intraclass Correlation Coefficient
ICF	Informed Consent Form
IRB	Institutional Review Board
ISI	Interstimulus Interval
ITT	Intent-To-Treat
MAST	Multimodal Automated Sensory Testing
NRS	Numerical Rating Scale
NSAID	Nonsteroidal Anti-Inflammatory Drug
OA	Osteoarthritis
PDN	Painful Diabetic Neuropathy
PGIC	Patient Global Impression of Change
PPT	Pressure Pain Threshold
SAE	Serious Adverse Event
SES	Standardized Effect Size
VAS	Visual Analog Scale

1. INTRODUCTION

1.1. Overview

Despite having validated methods for collecting pain measures that have been traditionally used, clinical trials of analgesics have been plagued by the insensitivity of pain outcome measures due in large part to the large variability associated with the subject's perception of pain and inconsistent reporting of these measurements. Large variability in data erodes assay sensitivity, which in turn, decreases statistical power. The practical impact of this large variability is that large numbers of participants are often used in order to sufficiently power an analgesic trial to detect significant efficacy of analgesics over placebo. Seemingly adequately powered studies of known analgesics routinely fail to separate from placebo.

The true failure rate of analgesic clinical trials is unknown and will not be known in the foreseeable future, due to (i) publication bias (failed studies are frequently unpublished); (ii) the absence of submission requirements of nonregulatory studies to regulatory authorities, where they could potentially contribute to analyses of failed studies; (iii) the frequent lack of active controls in analgesic trials, removing the possibility of firmly distinguishing failed studies (bad design/execution) from negative studies (the drug was ineffective); and (iv) sluggish or incomplete submission of study results to public trial postings.

It is unclear how many early studies failing to show efficacy have failed due to design issues. Even if study failure never occurred, sample size calculations based on the literature lead sponsors to include 50-60 participants per arm in analgesic studies, based on typical between-group treatment differences in mean pain intensity of about 1 point (0-10 scale) and typical standard deviations of these scores of about 2.5 points. Recruiting over 100 participants with chronic pain typically has required multicenter studies, introducing difficult-to-quantify inter-site variability in study conduct.

This high failure rate and use of large numbers of participants, particularly for early-stage development, are impediments to the development of new analgesics. Methods that can reduce the error variance in pain perception, measurement, and reporting could substantially reduce the number of participants required for these trials and could yield the amplifying advantage of allowing multicenter studies to be performed as single-center studies, further decreasing variability.

Participants in clinical trials of analgesics vary in the reliability with which they are able to report their pain experience in quantifiable terms (Quiton 2008). For example, a participant may give substantially differing numerical rating scale (NRS) scores between in-clinic visits but also verbally describe his or her pain as not having changed. Since it is impossible to know whether a change in report is due to poor reporting or actual change (as there is no objective standard measure to compare the participant's subjective report to) this is more easily demonstrated with painful stimuli of known quantities. This also allows researchers to quantify the participant's reporting ability. For example, a pressure stimulus known to be painful to the participant, say 2kg/cm^2, can be applied and rated by the participant multiple times. If the participant's NRS reports are very similar across trials they would be considered reliable; if the

patient's NRS reports are highly variable for a fixed stimulus, they would be considered unreliable.

Given that trial participants' ability to reliably report pain varies, the question arises: is this a skill that can be improved? It is known that the ability to reliably quantify stimuli can be improved by training in other sensory modalities such as the auditory, as in the case of studies training participants to provide more reliable reports across different study sites (Baumann 2004). We also believe, based on prior work comparing reporters with high variability reports of evoked pain to those with low variability, that reliable reporting does contribute to participants' ability to discriminate a known analgesic from placebo.

Participants' ability to accurately discriminate an analgesic from placebo may also be influenced by their prior experience with both types of treatment. A 1989 study by Fedele and colleagues found that patients responded less to placebo in successive rounds of treatment for dysmenorrhea (from a peak of 84% on the first round to only 10% by the fourth) (Fedele 1989). Anecdotal evidence from experienced researchers also suggests that some participants (identified via some unspecified heuristic method by study staff) are simply better able to discriminate between active and placebo treatments.

This combination of factual and anecdotal evidence has led us to hypothesize that training may improve participants' abilities and therefore trial assay sensitivity. The current study is designed to test two training paradigms against a control group using a known analgesic in order to determine whether training can in fact increase a group of patients' ability to accurately discriminate an active analgesic from placebo. One training paradigm, Evoked Pain Training (EPT) takes advantage of the fact that perceptual discrimination appears to be a learnable skill and trains participants to be better discriminators (connoisseurs) of their pain experiences. The other, Drug/Placebo Administration (DPA) training, delivers a variety of single-dose active drug and placebo experiences to the participant, with the goal of reducing response to placebo, loosely analogous to the Fedele study.

1.2. Study Design Rationale

1.2.1. General Design Rationale

The study is divided into two distinct stages.

The first stage, termed the Training stage, is a non-blinded, randomized parallel design in which participants will be randomized to one of three training conditions: EPT, DPA, or Control (C) (no special training). In this stage, participants and study staff will necessarily know which training group the participants are assigned to. Participants will also be aware of what the other training conditions are, as they must be fully informed of all possible conditions they might be assigned to prior to consent. The design is parallel in order to clearly distinguish the effect, if any, each training has without contamination or carry-over effects from another condition.

Within the DPA training arm participants will undergo a double-blind, randomized, 4-period crossover treatment wherein they receive 2 doses of placebo, 1 dose of oxycodone, and 1 dose of pregabalin in a series of four single-dose in-clinic visits. The order of treatments will be

randomized, but the participant will be unblinded after each dose (detailed below) for training purposes.

Within the EPT training arm participants will undergo weekly training sessions, one per week for four weeks. EPT includes evaluation of participants' pressure pain threshold and tolerance, matching pressure pain to index pain, repeated rating of randomized noxious stimuli (of intensities between threshold and tolerance) and feedback on consistency of ratings.

Control condition participants will receive no special training or experiences.

The second stage, Training Evaluation stage, is a double-blinded, placebo-controlled, randomized, 2-period crossover study. Randomization will be used to minimize bias in the assignment of participants to treatment sequences and to increase the likelihood that known and unknown subject attributes (e.g., demographic and baseline characteristics) are evenly balanced across treatment sequences. Blinded treatment will be used to reduce potential bias during data collection and evaluation of clinical endpoints. The crossover design is being used (i) to minimize subject variability (with each subject being used as his/her own control), and (ii) to minimize the number of participants needed for evaluations.

1.2.2. Treatments

Study Medications

A placebo is being used in order to establish the magnitude of changes in clinical endpoints that may occur in the absence of an active treatment and provide an adequate control for evaluating analgesic effect.

Pregabalin, an analgesic known to be effective in treating painful diabetic neuropathy (PDN) (Lesser 2004, Rosenstock 2004, Richter 2005), was chosen in order to evaluate the relative magnitude of any differences in the ability to detect a true analgesic effect. If an active treatment of unknown efficacy for the indication were used, we would have no way of knowing if a lack of difference were due to training having no effect or the treatment having no true effect. Oxycodone was chosen as one of the treatments during DPA training because it is a commonly used prescription analgesic with known efficacy and has a different side-effect profile than does pregabalin.

All treatments for the DPA Training stage will be over-encapsulated to be visually indistinguishable for each other. All treatments for the Training Evaluation stage will be over-encapsulated to be visually indistinguishable from each other. DPA treatments may or may not be distinguishable from Training Evaluation stage treatments. DPA and Training Evaluation are distinct, non-overlapping, stages; therefore it is not problematic if treatments between stages are distinguishable.

Treatment Duration

Total treatment duration of 10 days was chosen as the minimum duration necessary to demonstrate efficacy. This includes a 3 day titration period and 1 week of stable dose (an additional 3 days of titration is allowed for participants with tolerability issues). Previous studies have shown efficacy of pregabalin at 1 week (Lesser 2004, Rosenstock 2004). No taper

Asentral, Inc. Institutional Review Board approved – August 22, 2013

period is considered necessary due to the short duration of treatment and exclusion of patients with seizure disorders minimizing any adverse reaction to discontinuation. Since the primary motivation of the study is to test methodology and not drug efficacy, the shortest possible treatment period was selected to minimize patient burden.

Washout Duration

There is a 7 +/- 1-day washout period between the end of Treatment Period A and the beginning of Treatment Period B. There is no washout period prior to Treatment Period A or prior to the training stage. Participants on stable daily doses of opioids allowed by the protocol will be asked to refrain from taking any morning dose of opioids on the day of any DPA single-dose training visits (in order to avoid any potential cumulative effect with the oxycodone dose used in that training sequence).

2. STUDY OBJECTIVES

2.1. Primary Objective

- To assess the ability of EPT and DPA training to increase subjects' ability to discriminate between active and placebo treatments in a double-blind crossover trial of a known analgesic, measured by standardized effect size, relative to untrained control subjects.

2.2. Secondary Objectives

- To evaluate whether baseline characteristics of subjects predict response to training, measured by differences in psychophysical profile between baseline and end of study.

3. STUDY ENDPOINTS

The study endpoints are:

- Primary Endpoint 1 (Treatment Efficacy): Difference between treatment periods (pregabalin vs. placebo in Training Evaluation stage) in change from baseline 0-10 NRS 24h average pain intensity.
- Primary Endpoint 2 (Training Efficacy): Difference between treatment periods (pregabalin vs. placebo in Training Evaluation stage) and between training conditions (EPT, DPA training, and C) in change from baseline 0-10 NRS 24h average pain intensity.
- Safety Endpoint: Adverse events (AEs) reported.
- Secondary Endpoints:
    - Change from baseline 0-10 NRS current, 24-hour worst and walking pain intensity
    - Patient Global Impression of Change (PGIC)
    - Patient Preference for Treatment
    - Change from baseline in psychophysical function variables

Asentral, Inc. Institutional Review Board approved – August 22, 2013

- Perceptual power function intercept and slope exponent
- Evoked pain report reliability (coefficient of variation [CoV])

4. STUDY DESIGN

This is an investigator-initiated, exploratory, single-center, double-blind, randomized, placebo-controlled, crossover study of pregabalin versus placebo to evaluate methods of training participants to improve study assay sensitivity. The duration of participation will be up to 54 days. Eligible participants will have chronic pain as a result of diabetic neuropathy. After meeting initial entry criteria participants will be randomized to one of three parallel training conditions, EPT, DPA, or Control ("Training stage"). Participants in the EPT group will undergo 4 weeks of in-clinic training using the EPT paradigm in 4 sessions spaced approximately 1 week apart. In EPT participants will repeatedly rate an evoked pain delivered via pressure to the thumbnail and receive feedback. Participants in the DPA group will be administered randomized, double-blind, single-doses of placebo (twice), pregabalin (once), and oxycodone (once) in-clinic, rate their experience, and be unblinded after treatment. The C group will have no special training administered. Participants will be required to have a minimum pain score of at least 4 on the 0-10 NRS to be randomized into the Treatment Evaluation stage, which is a 2-period crossover study. After baseline assessments, they will be treated in a double-blinded fashion with either pregabalin or placebo for at least 10 days, including a 3-6 day titration period (pregabalin 150mg/day) and a 7-day stable treatment period (pregabalin 300mg/day) with 1 50-mg capsule (titration) or 2 50-mg capsules (stable treatment) tid. At the end of Treatment Period A, participants will have a 7 +/- 1 day washout period followed by 10- to 13-day Treatment Period B with the alternate treatment following the same procedure. Participants will be encouraged to maintain their customary level of physical activity during the study. Figure 1 illustrates the study design.

Figure 1. Study Design

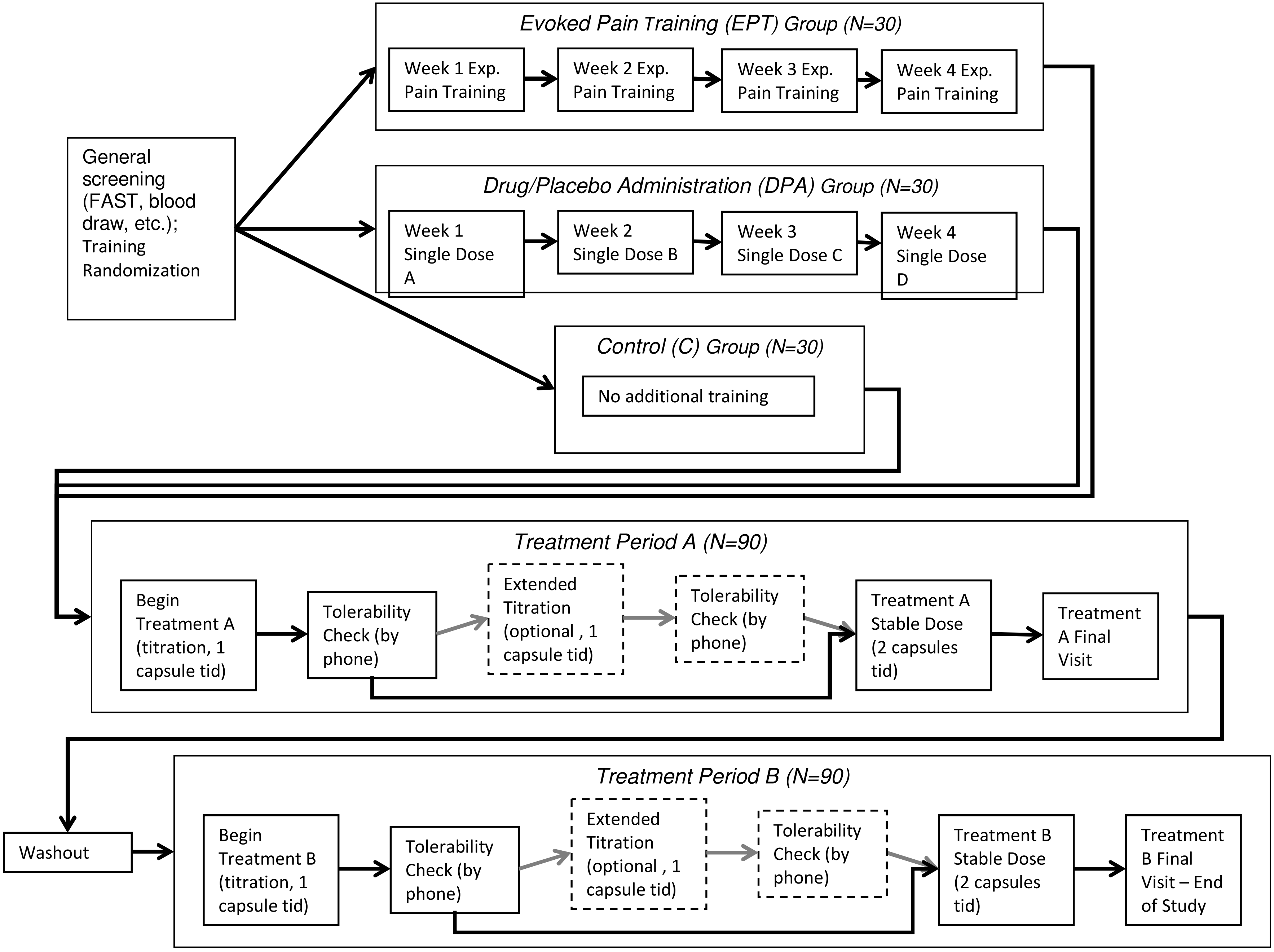

5. STUDY POPULATION

5.1. Number of Participants

Up to 105 participants will be randomized to the training conditions, intending 30 completers per arm with up to 5 replacements allowed for dropouts from each training condition.

Participants who discontinue early after being randomized to treatment in the Training Evaluation stage will not be replaced. Only 5 replacements will be made in each training condition arm; further dropouts beyond 5 in a training condition will not be replaced.

5.2. Duration of Study

Participants will be in the study for up to a maximum of 62days (68 days with optional extended titration), a period that includes an approximately 4-week training period, 10-day treatment (Treatment Period A), a 7 +/- 1 day washout period, and a 10-day treatment (Treatment Period B).

5.3. Number of Study Centers

One clinical center is planned.

5.4. Eligibility Criteria

5.4.1. Inclusion Criteria

A subject must meet all of the following criteria to be enrolled in the study:

1. Be a man or a non-pregnant, non-lactating woman 18 years or older. Women of childbearing potential should be willing to use an acceptable birth control method (at the Investigator’s discretion) during the study to avoid pregnancy.
2. Have voluntarily provided written informed consent (see attached ICF).
3. Be able to speak, read, write, and understand English, understand the consent form, complete study-related procedures, and communicate with the study staff.
4. Have a clinical diagnosis of Painful Diabetic Neuropathy (PDN) for at least 6 months.
    i. Clinical diagnosis may be verified by medical records or by clinical examination during the first visit combined with a medical history of appropriate symptoms for at least 6 months.
5. Have a pain intensity score averaging ≥4 on a 0-10 NRS for average daily recall over past 24 hours. (This applies at V1, V2, and V5.)
6. Have an average daily pain intensity of at least 4 on the 0-10 NRS on at least 20 out of the past 30 days.
7. Be, in the opinion of the Investigator, in sufficiently good health to participate in the study at screening, based upon the results of a medical history, physical examination and laboratory analysis.

Prior to each Treatment Period, the participants must meet the following additional criteria for randomization: Have average pain intensity (24-hour recall) ≥4 on the 0-10 NRS.

5.4.2. Exclusion Criteria

A subject must be excluded if any of the following criteria are met:

1. Are pregnant and/or lactating.
2. Have been diagnosed as having any inflammatory arthritis, gout, pseudo-gout, Paget's disease, fibromyalgia or any chronic pain syndrome that in the Investigator's opinion would interfere with the assessment or self-evaluation of pain and other symptoms of PDN.
3. Have evidence for multiple causes of pain in the neuropathic pain area, such as lumbar radiculopathy.
4. Have received or used any of the excluded/prohibited treatments or drugs specified in the list of prohibited treatments (below) or are unable to agree to the list of treatments prohibited during the study.
5. Have a history of congestive heart failure, unstable coronary artery disease, stroke, or uncontrolled hypertension.
6. Have a history of significant gastrointestinal disease, including active gastro-duodenal ulcerations, perforations, or bleeds.
7. Have abnormal clinical laboratory test results or vital signs unless deemed not clinically significant by the investigator.
8. Have regularly worn false fingernails within the past 6 months (more than 25% of the time)
9. Are undergoing active treatment for cancer, are known to be infected by human immunodeficiency virus, or are being acutely and intensively immunosuppressed following transplantation.
10. Have a history of alcohol or other substance abuse (not including nicotine or tobacco) within 5 years.
11. Have a history of suicide attempt within the past 1 year or suicidal ideation within the past 1 month.
12. Have a history of epilepsy or other seizure disorder.
13. Have creatinine clearance below 60 mL/min as calculated by Cockroft-Gault equation for serum creatinine.
14. Known to have a condition that in the Investigator's judgment precludes participation in the study.
15. Have previously been admitted to this study.
16. Are involved in a worker's compensation, disability claim, or litigation related to medical condition or treatment that is open or was settled within the past 12 months. (Whether litigation is related to medical condition or treatment may be decided at the Investigator's discretion. Claims settled >12 months previously are permitted.)

17. Have a known failure to respond to pregabalin, gabapentin, or oxycodone due to either efficacy or tolerability in previous treatment at therapeutically appropriate doses.
18. Are allergic to or have a hypersensitivity to pregabalin or oxycodone.

Prohibited treatments:

- Use of pregabalin or gabapentin within 2 weeks of the study and for the duration.
- Use of opioids in doses greater than 80mg morphine equivalent per day, either PRN or stable daily doses, within 1 week of study and for the duration of the study.
- Use of prescription capsaicin 8% patch (e.g., Qutenza®) within 3 months. (Over-the-counter topical capsaicin creams [0.025% and 0.075%] are not excluded.)
- Nerve block or intrathecal analgesia within 6 weeks of study.
- Neuro-ablation or neurosurgical intervention for their PDN.
- Any investigational drug or have used an investigational device in the 30 days prior to study entry.
- As-needed (PRN) use of NSAID or opioid compounds (oral or topical) for the duration of the Training Evaluation stage of the study. NSAID or opioids taken at a stable dose and consistent daily regimen are permitted.

The following concomitant medication rules apply specifically to participants in the EPT and DPA training conditions:

Participants in the EPT or DPA training are permitted the use of PRN analgesic medications during the Training stage of the study, provided that they agree not to take any PRN medication on the day of a training visit prior to the visit. PRN analgesic use after completion of a training visit is permitted.

The following concomitant medications are specifically allowed:

- Concomitant medications not specifically excluded are permitted, including analgesics, if they are at stable doses taken on a regular schedule at the Investigator's discretion.
- Use of sliding scale insulin is specifically permitted during the study.

Time and Events Schedule of Procedures is detailed below, divided by study stage

Table 1. Screening/Baseline Study Procedures

**Visit**	**V1**
**Clinic visit (C) or telephone call (T)**	**C**
**Study day(s)[a]**	**1**
Informed Consent	X
Inclusion/Exclusion criteria	X
Urine pregnancy test	X
Medical History/ Demographics/ Previous Treatment Experience	X
Vital Signs (BP & HR)	X
General Physical Exam	X
Blood Draw (For creatinine clearance)	X
Pain Intensity Ratings (0-10 NRS)	X
Focused Analgesia Selection Task (FAST)	X
Randomization to training condition[1]	X

[1] Randomization to training condition will occur 1-2 days after screening visit, upon confirmation of creatinine clearance results.

Asentral, Inc. Institutional Review Board approved – August 22, 2013

Evoked Pain Training

**Visit**	**T1**	**T2**	**T3**	**T4**
**Study day(s)**	**2**<br>**(+/- 3 days)**	**9**<br>**(+/- 3 days)**	**16**<br>**(+/- 3 days)**	**23**<br>**(+/- 3 days)**
**MAST pressure training and feedback**	X	X	X	X
**Pain Intensity Ratings (0-10 NRS)**	X	X	X	X
**Training Feedback Questionnaire**				X

Drug/Placebo Administration (DPA) Training

**Visit**	**T1**	**T2**	**T3**	**T4**
**Study day(s)** [a, b]	**2**<br>**(+/- 3 days)**	**9**<br>**(+/- 3 days)**	**16**<br>**(+/- 3 days)**	**23**<br>**(+/- 3 days)**
Pain Intensity Ratings<br>(0-10 NRS, at hours 0, 2 and 4)	X	X	X	X
PGIC<br>(at hour 4)	X	X	X	X
Vital signs<br>(BP & HR, hours 0, 2, 4)	X	X	X	X
Treatment Experience Questionnaire (TEQ)	X	X	X	X
Unblind Treatment, discuss with patient<br>(after all other assessments at hour 4)	X	X	X	X
Treatment	1 capsule	1 capsule	1 capsule	1 capsule
AE Assessment<br>(hours 2 and 4)	X	X	X	X
Training Feedback Questionnaire				X

EPT and DPA sessions will be scheduled 1 per week for 4 weeks; timing is flexible but visits must be separated by 7 +/-3 days (min. 4, max 10)

Asentral, Inc. Institutional Review Board approved – August 22, 2013

Control Group

No additional training – immediately begin Training Evaluation Stage

Asentral, Inc. Institutional Review Board approved – August 22, 2013

Training Evaluation Period

	Treatment Period A						Treatment Period B							End of Study
	Visit 2a	Visit 2b[1] *(optional)*	Visit 3a	Visit 3b[2] *(optional)*	Treatment Period (At Home)	Visit 4	Washout	Visit 5a	Visit 5b[1] *(optional)*	Visit 6a	Visit 6b[2] *(optional)*	Treatment Period (At Home)	Visit 7	Visit 8
	Day 30<br>+/- 3 days	+/- 3 days	Day 32		Day 33-39 (or 40)<br>7-8 Days	Day 40<br>+/- 3 days	Days 40-46	Day 47<br>+/- 3 days		Day 49		Day 50-56 (or 57)<br>7-8 Days	Day 57<br>+/- 3 days	Day 64<br>+/- 3 days
	In Clinic<br>Begin Treatment	In Clinic	Telephone<br>Tolerability Check	Telephone<br>Tolerability Check #2		In Clinic<br>End Treatment		In Clinic<br>Begin Treatment	In Clinic	Telephone<br>Tolerability Check	Telephone<br>Tolerability Check #2		In Clinic<br>End Treatment	Telephone
**Assessments**														
**Treatment Randomization**	X													
**Vital Signs**	X	X				X		X	X				X	
**Urine Pregnancy Test**	X													
**Pain Intensity Ratings (0-10 NRS)**	X	X				X		X	X				X	
**PGIC**						X							X	
**Tolerance Check** *(Investigator Discretion)*			X	X						X	X			
**Patient Global Preference for Treatment**													X	
**Focused Analgesia Selection Task (FAST)**														
**AE Assessment**	<--- Throughout Study --->													
**ConMeds Assessment**	<--- Throughout Study --->													

Asentral, Inc. Institutional Review Board approved – August 22, 2013

[1] Subjects will be allowed to return once if they fail the pain assessment at the first visit.
[2] An additional 3 days titration will be allowed per investigator discretion.

Study visits for C participants are minus 28 days (the approximate duration of training for the other conditions). Optional extended titration periods may add 3 days to each treatment period.

Maximum study duration is 68 days with extended titration in both treatment period. Total duration will be approximately 28 days shorter for participants in the C condition and may be shorter for participants in the EPT or DPA conditions depending on exact scheduling of training sessions.

5.5. Screening - Visit 1 (Day 1)

Participant recruitment will be conducted by the Investigators and/or staff at the clinical site. Potential participants will be recruited by local advertising approved by the Institutional Review Board (IRB). During the screening and recruitment process, the Investigators will be responsible for describing the nature of the clinical study, verifying that the eligibility criteria have been met, and obtaining informed consent.

The following specific procedures will be conducted and documented:

5.5.1. Informed Consent

All participants will provide written informed consent for the study prior to collection of study data or performance of study procedures/treatment Assignment of Subject Number

To de-identify participants' information, a unique identification number will be given to all participants who provide written informed consent.

All participants who provide informed consent will be given a 5-digit number. The first 2 digits will be the clinical site number, with 01 being clinical site 1. The last 3 digits will be numbers of 001 to 999 assigned in ascending sequential order during the screening visit.

5.5.2. Eligibility

The subject's eligibility for study enrollment will be reviewed and documented and will include the following:

- Demographic Information: The participant's demographic information will be documented on the appropriate CRF and will include date of birth, gender, height, weight, body mass index (BMI) (calculated; see Appendix 15.8), race, and ethnicity.
- Previous Study and Treatment Experience: The number of previous clinical drug studies that the participant has been treated in, the total duration of time spent in such studies, and the number of previous drug treatments used to treat PDN (in clinical studies or outside of studies) will be recorded.
- Medical History: Recent and relevant medical history will be obtained for the past 3 years. Medical history relating to diabetes and painful neuropathy will be obtained from the diagnosis of diabetes to present.
- Prior and Concomitant Medications: All medications currently being taken and those taken within the past year will be documented as completely as possible.
- Non-fasted Clinical Laboratory Tests: Blood samples for serum chemistry will be collected. The following tests will be performed by the local laboratory.
    - Serum Chemistry Panel
        - Creatinine
- Urine Pregnancy Test

Asentral, Inc. Institutional Review Board approved – August 22, 2013

A urine sample for pregnancy test will be collected (women of child bearing age only).

- Physical Examination: A brief physical exam will be conducted, and the PDN diagnosis confirmed. The study Investigator or authorized designee (who must be a physician, physician's assistant or nurse practitioner) will perform the physical examinations. Height and body weight will be measured at Screening Visit 1 only.
- Vital Signs: Sitting blood pressure and heart rate measurements will be assessed with a completely automated device consisting of an inflatable cuff and an oscillatory detection system. All values will be registered on a built-in recorder so that measurements are observer independent. Blood pressure and heart rate measurements will be assessed while the subject is in the sitting position. Manual blood pressure readings may be obtained in the event of instrument malfunction.

All enrollment criteria will be reviewed to ensure that participants meet all inclusion and none of the exclusion criteria to the extent possible. Note: laboratory values may be reviewed at the next visit.

5.5.3. Clinical Pain Intensity

All participants will be asked to rate the following on a 0 to 10 NRS scale, where 0 is "No Pain" and 10 is "Worst Pain Imaginable":

- Current Pain Intensity from PDN
- Average Pain Intensity from PDN in the past 24 hours
- Worst Pain Intensity from PDN in the past 24 hours
- Pain on Walking in the past 24 hours

5.5.4. Focused Analgesia Selection Task (FAST)

All participants who meet all preliminary entry criteria will undergo the FAST assessment procedures as described in Appendix 15.1. Note: No feedback or training will be provided after the psychophysical assessment portion.

5.5.5. Training Assignment

For participants who meet all entry criteria and are randomized into the training stage of the study, a training condition number will be assigned and will consist of 3 digits, 001-105. Training condition number is distinct from the subject ID number. Training condition numbers will be assigned randomly by the statistician. Subjects will not be randomized to a training condition until all eligibility criteria are confirmed, including lab results.

Note: Participants who discontinue the study during the Training Stage but prior to treatment randomization in the Training Evaluation Stage will be replaced up to a maximum of five (5) participants in each training condition. Participants discontinuing

after treatment randomization will not be replaced and the sixth or subsequent participants discontinuing during the Training Stage will not be replaced.

For each training condition number 001-105, the assignment to training condition (EPT, DPA, or C) will have been determined in accordance with the pre-determined randomization scheme prior to study start. This will have been done in blocks to ensure that approximately equal numbers of participants are assigned to the 3 training conditions on an ongoing basis. There will be 30 participants in each of the three training conditions.

Assignment to training condition is NOT blinded. The participant and Investigator will know which training condition the participant is assigned to.

5.6. Training Stage

5.6.1. Evoked Pain Training Condition - Visits T1 through T4 (Days 2 through 29)

Participants assigned to the EPT condition will be scheduled for 4 training visits. For simplification of visit schedules between training and control conditions training visits will be designated Visits T1 through T4. Training visits will be scheduled approximately 1 week apart with the following conditions:

- Visit T1 (first training visit) must be at least 1 day after Screening
- Visits must be at least 4 days apart, but not more than10 days apart

At the end of the last training visit, participants will be administered the Participant Training Feedback Questionnaire (see Appendix 15.7).

5.6.1.1. Evoked Pain Training

At each training visit (T1 through T4) participants assigned to the EPT condition will undergo EPT as described in Appendix 15.2, including threshold and tolerance assessment, rating of index pain, and psychophysical profiles with feedback.

During the 3-4 week EPT period participants will be allowed to continue the use of any medications allowed under the study inclusion/exclusion criteria, including morning doses of permitted analgesics.

5.6.2. Drug/Placebo Administration Training – Visits T1 through T4 (Days 2 through 29)

Participants assigned to the DPA condition will be scheduled for 4 training visits. For simplification of visit schedules between training and control conditions training visits will be designated Visits T1 through T4. Training visits will be scheduled approximately 1 week apart with the following conditions:

- Visit T1 (first training visit) must be at least 1 day after Screening
- Visits must be at least 4 days apart, but not more than10 days apart

The use of concomitant medications allowed by the study inclusion/exclusion is permitted between training visits. If the participant is on stable and regular doses of any opioid as

permitted by the protocol they must not take any opioid during the day of a DPA training visit prior to the visit. Every effort will be made to schedule DPA training visits for the AM.

5.6.2.1. DPA Treatment Randomization

At the first DPA training visit (T1) participants assigned to the DPA condition will be randomized to a treatment sequence for their DPA treatments. Note: this is distinct and fully independent from their treatment assignment in the Training Evaluation stage of the study. A DPA treatment assignment number will be assigned at the first DPA training visit, T1.

DPA participants will be assigned a six digit DPA sequence number formatted as ##-#-###. The first two digits are the site number, and will always be 01, as there is only one site participating in the study; The second digit is the # of the week of treatment, (1-4; the last three digits are a randomly assigned subject number from 001 to 035. For each DPA sequence number the assignment to treatment sequence will have been determined in accordance with the pre-determined randomization scheme prior to study start. Treatment sequences will be randomly determined among the four treatments: oxycodone 10mg, pregabalin 150mg, and two placebo treatments. Note: participants will have been informed that they will be receiving a randomly assigned series of four treatments that may include oxycodone, pregabalin, or placebo. They will not have been told how many of each treatment are in the sequence in order to minimize their ability to determine the content of their last treatment by process of elimination.

5.6.2.2. Pre-treatment Assessments

5.6.2.3. Vital Signs:

Sitting blood pressure and heart rate measurements will be assessed with a completely automated device consisting of an inflatable cuff and an oscillatory detection system. All values will be registered on a built-in recorder so that measurements are observer independent. Blood pressure and heart rate measurements will be assessed while the subject is in the sitting position. Manual blood pressure readings may be obtained in the event of instrument malfunction.

5.6.2.4. Clinical Pain Intensity:

All participants will be asked to rate the following on a 0 to 10 NRS scale, where 0 is “No Pain” and 10 is “Worst Pain Imaginable”:

- Current Pain Intensity from PDN
- Average Pain Intensity from PDN in the past 24 hours
- Worst Pain Intensity from PDN in the past 24 hours
- Pain on Walking in the past 24 hours

Participants’ current pain intensity from PDN must be at least 4 on the 0-10 NRS in order to continue with a DPA training visit. If their current pain is below 4 their training visit may be

rescheduled within 1 week. This may be done up to two times for a given participant (over the entire duration of the DPA training period). If a participant fails to meet the current pain criterion a third time they must be discontinued, but may be replaced according to the protocol conditions for replacement. Note that this may extend the total duration of enrollment for a DPA participant up to an additional 14 days if they reschedule two DPA training visits at the maximum allowed intervals.

5.6.2.5. DPA Training Stage Treatment

Authorized clinical staff will remove the assigned treatment medication from the blister pack. Participant will self-administer the medication under supervision of the clinic staff. At 2 hours post-treatment vital signs will be taken and participants will rate their Current Pain Intensity from PDN on the 0-10 NRS.

5.6.2.6. Post-treatment Assessment and Feedback

At 4 hours after treatment vital signs will be taken a final time and participants will:

- Rate Current Pain Intensity from PDN on the 0-10 NRS
- Rate PGIC
- Answer the Treatment Experience Questionnaire (see Appendix 15.3)

After providing ratings, the clinic staff will partially unblind the treatment just received to the participant, telling them whether the treatment was an active drug or a placebo. The specific treatment will not be unblinded, only whether it was active or placebo. Clinic staff will then review the participant's responses to the Treatment Experience Questionnaire with the participant with particular attention paid to the reasons why the participant believed they received drug/placebo. For example, if the participant believed they received active drug because of a perceived side effect but actually had placebo, the clinician might emphasize to the participant that it is possible to experience side effects even when on placebo. Conversely, if the participant is confident that they received active drug because they experienced meaningful pain relief after actually receiving active drug, this strategy would be endorsed.

Note: All reasonable efforts will be made to prevent the clinic staff or participants from being unblinded to treatment prior to intentional unblinding, particularly by process of elimination at the final visit. To this end, participants will not be told how many of each type of treatment they will be receiving, only that during the DPA training they will be given a randomized sequence that may include pregabalin, oxycodone, and placebo. Records of previous visits' unblinding will not be apparent in the materials available to the clinic staff during the visit. Whenever possible the end-of-visit unblinding and debrief will be conducted by different clinical staff members such that no one person unblinds a participant at all four DPA training visits. It is acknowledged that the double-blind during DPA training may not be perfect. For example, a single clinic staff member sees a given participant at all four visits due to scheduling issues and might conceivably remember that the participant's previous three visits included only one placebo and conclude that the final

visit must be a placebo as well. However, since the blinding for DPA training sessions is not critical for any test of drug safety or efficacy this is considered acceptable.

Participants must meet minimum clinical discharge criteria before being released from the clinic at the conclusion of each visit.

At the end of the last training visit, participants will be administered the Participant Training Feedback Questionnaire (see Appendix 15.7).

5.6.3. Control Training Condition

Participants assigned to the C condition for training will receive no training and will not make any in-clinic training visits corresponding to T1 through T4 as EPT and DPA condition participants do.

Participants in the C condition may proceed to the Training Evaluation stage immediately. However, V2 should not be scheduled prior to confirmation of all eligibility criteria including creatinine clearance and scheduling of Visit 2 should be as close as possible to 7 days from Visit 1.

5.7. Training Evaluation Stage

5.7.1. Visit 2 (Begin Treatment Period A)

Note: Training Evaluation stage begins with Visit 2. Participants withdrawing from the study prior to Visit 2 will be replaced, up to 5 in each training condition. Participants withdrawing or discontinued from the study at Visit 2 or later will not be replaced.

All reasonable efforts should be made to schedule Visit 2 approximately 1 week after the conclusion of the training period for participants in the EPT and DPA conditions.

5.7.1.1. Review of Eligibility Criteria

Review all inclusion/exclusion criteria, including the following:

- Vital signs
- In-clinic pain NRS (24-hour recall) to ensure subject meets minimum pain requirement of 4 on the 0-10 NRS

Participants who meet all eligibility for randomization, including minimum pain intensity scores, will continue in the study. Those who fail to meet eligibility criteria will be discontinued and will not be replaced, with the exception that if a participant does not meet the minimum 24h average pain intensity requirement they may be rescheduled to attempt Visit 2 within 1 week. Participants failing to meet the minimum pain intensity criterion twice in a row will be discontinued and not replaced.

5.7.1.2. Treatment Randomization

For participants who continue to meet all criteria and are randomized into the Training Evaluation stage of the study, a treatment number will be assigned and will consist of 3

digits, starting with 201 to 305. Treatment number is distinct from subject ID number or training condition number. Treatment condition numbers will be assigned as participants enter the Training Evaluation stage and after confirmation of eligibility. Forty-five (45) participants will be assigned pregabalin as Treatment A with placebo as Treatment B and 45 participants will have placebo as Treatment A with pregabalin as Treatment B. Within each treatment order 15 participants will have come from each of the three training conditions, as shown in Table **2** Training/Treatment Randomization.

Table 2 Training/Treatment Randomization

	**Treatment Condition (A-B)**	
Training Condition	Pregabalin-Placebo	Placebo-Pregabalin
EPT	n=15	n=15
DPA	n=15	n=15
C	n=15	n=15

Note that the 2-digit treatment number assigning the pregabalin/placebo sequence for the training evaluation stage (cross-over) is not to be confused with the 3-digit training condition number assigning EPT/DPA/C condition (parallel).

5.7.1.3. Baseline A Assessments

5.7.1.4. Vital Signs

Blood pressure and heart rate measurements will be assessed while the subject is in the sitting position in the same manner as at Visit 1.

5.7.1.5. Pain Intensity

All participants will be asked to rate the following on a 0 to 10 NRS scale, where 0 is “No Pain” and 10 is “Worst Pain Imaginable”:

- Current Pain Intensity from PDN
- Average Pain Intensity from PDN in the past 24 hours
- Worst Pain Intensity from PDN in the past 24 hours
- Pain on Walking in the past 24 hours

Asentral, Inc. Institutional Review Board approved – August 22, 2013

5.7.1.6. Dispensing of Study Medication

Study medications for Treatment Period A will be dispensed according to the assigned randomization number. The study drugs will consist of capsules packaged in blister packages labeled for morning, mid-day and evening doses. Sufficient doses will be provided on each blister card for 3 days of titration at 1 capsule tid (inclusive of the day of Visit 2) followed by 8 days of stable dosing at 2 capsules tid to allow for some flexibility in scheduling. An additional 3 days of 1 capsule tid will be provided to accommodate an optional additional 3 days of titration if necessary (See Visit 3, Titration Check below for details of optional extended titration period). The capsules for both pregabalin and placebo will be identical looking, and both placebo and pregabalin will be packaged in identical-looking blister cards. The blister cards will contain no identifying information other than subject number, Treatment Period designation (A or B), and dosing instructions.

5.7.1.7. Dosing

Participants will be provided with Treatment Period A study medications for their treatment randomization number.

- **Initial Dosing:** The first dose of study medications is to be taken in the clinic after completion of all procedures for Visit 2. The subject will be instructed to take the evening doses for that day at least 6 hours later.
- **Dosing Schedule:** Dosing during the treatment periods will be three times per day dosing (tid). Dosage will be 1 capsule per dose during the titration period until tolerability is confirmed at Visit 3. After Visit 3 dosage will increase to 2 capsules per dose.
- **Methods of Administration:** All doses will be taken orally with a small glass of water or milk. The capsules may be taken with food. Participants should be informed that if they feel nauseated after taking the dose, their subsequent doses should be taken with food or milk.

5.7.1.8. Participant Instructions

Participants will be provided with dosing instructions and especially reminded not to increase dosing to 2 capsules until directed to (Visit 3 tolerability check).

5.7.2. Visit 3 (Tolerability Check)

Visit 3 is conducted by telephone at a scheduled time 3 days after Visit 2. Clinic staff will contact the participant by telephone to confirm tolerability of study medication.

If the participant is tolerating the medication acceptably well they will be instructed to begin dosing with 2 capsules tid for 7 days beginning the morning of the next day.

If the participant is clearly not tolerating medication they may be withdrawn at this point due to non-tolerability at the Investigator's discretion.

If the participant is having tolerance issues, they may be allowed an additional 3 days of titration at 1 capsule tid, at the Investigator's discretion considering the best interests of the participant. In this case a second Visit 3 tolerability check telephone call will be scheduled in 3 days. At this second titration check the participant will be instructed to either begin 2 capsule tid dosing as above if tolerating well or withdrawn due to non-tolerability as described above.

5.7.3. Visit 4 (End of Treatment Period A)

Visit 4 will be scheduled 10 days after Visit 2 (or 13 days if titration is extended). Participants will return to the clinic to be evaluated for the end of Treatment Period A.

5.7.3.1. End of Treatment Period A assessments

5.7.3.2. Vital Signs

Blood pressure and heart rate measurements will be assessed while the subject is in the sitting position in the same manner as at Visit 1.

5.7.3.3. Pain Intensity

All participants will be asked to rate the following on a 0 to 10 NRS scale, where 0 is "No Pain" and 10 is "Worst Pain Imaginable":

- Current Pain Intensity from PDN
- Average Pain Intensity from PDN in the past 24 hours
- Worst Pain Intensity from PDN in the past 24 hours
- Pain on Walking in the past 24 hours

5.7.3.4. PGIC

Participants will provide a rating of Patient Global Impression of Change (PGIC) from the beginning of Treatment Period A to the end of Treatment Period A.

5.7.4. Washout

After completing Treatment Period A (i.e., at the end of Visit 4) participants will enter a 7 +/- 1-day washout period prior to Treatment Period B. During the washout period any stable concomitant medications allowed by the protocol should be continued in the same fashion.

5.7.5. Visit 5 (Begin Treatment Period B)

Visit 5 to begin Treatment Period B will be scheduled 7 days after Visit 4. Visit procedures are identical to Visit 3.

5.7.5.1. Review of Eligibility Criteria

Review all inclusion/exclusion criteria, including the following:

- Vital signs
- In-clinic pain NRS (24-hour recall) to ensure subject meets minimum pain requirements

Participants who meet all eligibility for randomization, including minimum pain intensity scores, will continue in the study. Those who fail to meet these eligibility criteria will be discontinued and will not be replaced. If a participant does not meet the minimum 24h average pain intensity requirement they may be rescheduled to attempt Visit 5 within 1 week. Participants failing to meet the minimum pain intensity criterion twice in a row will be discontinued and not replaced.

5.7.5.2. Baseline B Assessments

5.7.5.3. Vital Signs

Blood pressure and heart rate measurements will be assessed while the subject is in the sitting position in the same manner as at Visit 1.

5.7.5.4. Pain Intensity

All participants will be asked to rate the following on a 0 to 10 NRS scale, where 0 is "No Pain" and 10 is "Worst Pain Imaginable":

- Current Pain Intensity from PDN
- Average Pain Intensity from PDN in the past 24 hours
- Worst Pain Intensity from PDN in the past 24 hours
- Pain on Walking in the past 24 hours

5.7.5.5. Dispensing of Study Medication

Study medications for Treatment Period B will be dispensed according to the assigned randomization number. The study drugs will consist of capsules packaged in blister packages labeled for morning, mid-day and evening doses. Sufficient doses will be provided on each blister card for 3 days of titration at 1 capsule tid followed (inclusive of the day of Visit 5) by 8 days of stable dosing at 2 capsules tid to allow for some flexibility in scheduling. An additional 3 days of 1 capsule tid will be provided to accommodate an optional additional 3 days of titration if necessary (See Visit 6, Titration Check below for details of optional extended titration period). The capsules for both pregabalin and placebo will be identical looking, and both placebo and pregabalin will be packaged in identical-looking blister cards. The blister cards will contain no identifying information other than subject number, Treatment Period designation (A or B), and dosing instructions.

5.7.5.6. Dosing

Participants will be provided with Treatment Period B study medications for their treatment randomization number.

- **Initial Dosing:** The first dose of study medications is to be taken in the clinic after completion of all procedures for Visit 5. The subject will be instructed to take the evening doses for that day at least 6 hours later.
- **Dosing Schedule:** Dosing during the treatment periods will be three times per day dosing (tid). Dosage will be 1 capsule per dose during the titration period until tolerability is confirmed at Visit 6. After Visit 6 dosage will increase to 2 capsules per dose.
- **Methods of Administration:** All doses will be taken orally with a small glass of water or milk. The capsules may be taken with food. Participants should be informed that if they feel nauseated after taking the dose, their subsequent doses should be taken with food or milk.

5.7.5.7. Participant Instructions

Participants will be provided with dosing instructions and especially reminded not to increase dosing to 2 capsules until directed to (Visit 6 tolerability check).

5.7.6. Visit 6 (Tolerability Check)

Visit 6 is conducted by telephone at a scheduled time 3 days after Visit 5. Clinic staff will contact the participant by telephone to confirm tolerability of study medication.

If the participant is tolerating the medication acceptably well they will be instructed to begin dosing with 2 capsules tid for 7 days beginning the morning of the next day.

If the participant is clearly not tolerating medication they may be withdrawn at this point due to non-tolerability at the Investigator's discretion.

If the participant is having tolerance issues, they may be allowed an additional 3 days of titration at 1 capsule tid, at the Investigator's discretion considering the best interests of the participant. In this case a second Visit 5 tolerability check telephone call will be scheduled in 3 days. At this second titration check the participant will be instructed to either begin 2 capsule tid dosing as above if tolerating well or withdrawn due to non-tolerability as described above.

5.7.7. Visit 7 (End of Treatment Period B)

Visit 7 will be scheduled 10 days after Visit 5 (or 13 if titration is extended). Participants will return to the clinic to be evaluated for the end of Treatment Period B and conclusion of the study.

5.7.7.1. End of Treatment Period B Assessments

5.7.7.2. Vital Signs

Blood pressure and heart rate measurements will be assessed while the subject is in the sitting position in the same manner as at Visit 1.

5.7.7.3. Pain Intensity

All participants will be asked to rate the following on a 0 to 10 NRS scale, where 0 is "No Pain" and 10 is "Worst Pain Imaginable":

- Current Pain Intensity from PDN
- Average Pain Intensity from PDN in the past 24 hours
- Worst Pain Intensity from PDN in the past 24 hours
- Pain on Walking in the past 24 hours

5.7.7.4. PGIC

Participants will provide a rating of PGIC from the beginning of Treatment Period B to the end of Treatment Period B.

5.7.7.5. Patient Global Preference for Treatment

Participants will answer a Patient Global Preference for Treatment question indicating which of the two treatments (Period A or Period B) they preferred.

5.7.7.6. FAST or Psychophysical Profile

Participants will complete the FAST assessment procedures as described in Appendix 15.1. Note: No feedback or training will be provided after the psychophysical assessment portion.

5.7.8. Visit 8, End of Study

Visit 8 will scheduled 7 days (+/- 3 days) after Visit 7.Participants will be called for an end of study safety check for Adverse Events.

5.7.9. Withdrawal and/or Early Termination Procedures

No follow-up visits will be scheduled, but participants with clinically significant AEs should be followed until satisfactory resolution.

A participant can be withdrawn from the study at the discretion of the Investigator for medical reasons or if the participant wishes to terminate the study. If a participant does not return for a scheduled visit, every effort should be made to contact the participant and to document the subject outcome, if possible.

Participants are considered lost to follow-up if they do not return to the office for scheduled visits to complete the study. Documentations of attempts to contact the subject must be included on the End of Study Form.

5.7.10. Unblinding of Subject Treatment

In the case of a medical emergency or in the event of a serious medical condition (such as a serious AE [an SAE]) when knowledge of the investigational product is essential for the clinical management or welfare of the subject, the Principal Investigator or other physician

managing a study subject may decide to unblind that subject's treatment code. The Investigator will record the date and reason for revealing the blinded treatment assignment for that subject in the appropriate CRF form.

6. STATISTICAL METHODS

6.1. Study Hypothesis

(a) Pregabalin will provide more pain relief (a greater difference between baseline and end of treatment) on the primary endpoint than placebo.

(b) There will be a greater difference between placebo and pregabalin for participants in the DPA condition than those in the C condition.

(c) There will be a greater difference between placebo and pregabalin for participants in the EPT condition than those in the C condition.

Additionally, given the exploratory nature of the study we anticipate evaluating without formal hypotheses which, if any, baseline participant characteristics are predictive of change in pain reporting characteristics (from psychophysical profile) for each training group.

6.2. Study Populations

Safety Population: This population will include all participants who receive at least 1 dose of study drug (placebo or pregabalin).

Per-protocol Population: The per-protocol population will include all participants who complete the 2 treatment periods without major protocol violation.

Intent-to-treat (ITT) Population: The intent-to-treat population will include all participants who receive at least 1 dose of study drug in both treatment periods.

6.3. Sample Size

A total of 105 participants are planned, targeting 90 participants in the Training Evaluation stage and allowing for replacement of up to 5 participants per training arm (15 total) to accommodate training attrition, bringing the total N to up to 105 participants.

Given the exploratory nature of the training programs, the effect size of any difference between groups is unknown. However, the interaction term of an analysis of variance (ANOVA) for 3 conditions by 2 treatments will provide a test of whether training group interacted with the difference between conditions. If we assume testing for marginal significance in this exploratory work (alpha=.10) and a Cohen's *f* of .15 (a medium effect for this measure) a sample of N=90 in 3 groups with 2 measurements provides a power of approximately .80.

Asentral, Inc. Institutional Review Board approved – August 22, 2013

Ninety participants also provides sufficient power for the entire sample (across training conditions) to differentiate between placebo and pregabalin. Assuming a moderate effect size of pregabalin in PDN of .35[c] a paired-comparison t-test between means (placebo vs. pregabalin) with alpha=.05 and power=.90 power analysis yields a sample size requirement of N=88. A sample of N=90 allows for convenient balancing of assigned conditions and treatment orders to six cells (3 training conditions and 2 treatment orders).

6.4. Randomization and Blinding

Randomization will be used to avoid bias in the assignment of participants to training condition and treatment sequence and to increase the likelihood that known and unknown subject attributes (e.g., demographic and baseline characteristics) are evenly balanced across the different design cells. Blinded treatment will be used to reduce potential bias during data collection and evaluation of clinical endpoints. During the double-blind period of the study, the subject and all personnel involved with the conduct and the interpretation of the study, including the Investigators, study center personnel, and the Sponsor (or designee) staff, will be blinded to the medication codes. Randomization data will be kept strictly confidential, filed securely by the Sponsor (or designee), and accessible only to authorized persons per Sponsor's (or designee's) standard operating procedures until the time of unbinding. In the event that an emergency unblinding is required, authorized/approved randomization system users, at the study centers and Sponsor (or designee), will have the ability to retrieve subject treatment groups assignment through the randomization system. Unblinding a subject should only be done in emergency situations for reasons of subject safety. The Investigator/study center should make every attempt to contact the Sponsor's or designee's Medical Monitor before breaking the blind. When the blinding code is broken, the reason must be fully documented. Refer to section 9.6.6 for further details of unblinding procedures specific to the different stages of the study.

6.5. Data Analysis

6.5.1. General Considerations for Efficacy Analysis

All efficacy analyses will be performed for all participants who complete the study without major protocol violations. If more than 10% of subjects randomized to Training Evaluation treatment are excluded for major protocol violations efficacy analyses will be repeated using the ITT population. Unless otherwise specified, all efficacy parameters will be presented by treatment group and by training condition group using summary statistics. Statistical analyses using analysis of variance (ANOVA) with repeated measures that are appropriate for cross-over design will be performed to compare treatments regarding

[c] This is slightly lower than some published studies to allow a margin for the short treatment period.

efficacy endpoints. Additional between-subject variables for training condition will be used where appropriate. Analyses of efficacy endpoints will be 2-sided with significance level of 0.05.

The primary efficacy endpoint, difference between treatment periods (pregabalin vs. placebo in Training Evaluation stage) in change from baseline 0-10 NRS 24h average pain intensity will be analyzed by a paired-sample comparison t-test. Mean between-group difference in pain intensity change from baseline for 24-hour worst and average, PGIC, and allodynia will be secondary outcome measures similarly analyzed by paired-sample comparison tests. These tests will be conducted to quantify the ability of the current study design to detect the efficacy of a known analgesic irrespective of the experimental training.

6.5.2. Methodology to Achieve Study Objectives[d]

Primary Objective:

- To compare the ability of Evoked Pain Training (EPT) and Drug/Placebo Administration (DPA) training to increase subjects' ability to discriminate between active and placebo treatments in a double-blind crossover trial of a known analgesic, measured by standardized effect size, relative to untrained control subjects.

Secondary Objective:

- To evaluate whether baseline characteristics of subjects predict response to training, measured by differences in psychophysical profile between baseline and end of study.

To compare the sensitivity of participants in different training conditions in discriminating the efficacy of pregabalin versus placebo:

Two methods will be used to address the primary objective of determining whether either training condition (EPT or DPA) provides superior assay sensitivity compared to the control condition on the primary endpoint of change in 24h average pain NRS.

1). Standardized effect size (SES) for each training condition will be determined using Cohen's d formula for SES (delta between conditions divided by pooled standard

[d] Note: Comparison of pregabalin vs. placebo across training groups is specified as a primary endpoint. However, testing the efficacy of this known analgesic is not a primary objective of the study and is therefore not listed here. The pregabalin vs. placebo comparison across all training groups will be used as an overall indication of how well the study design performed regardless of the training manipulation.

deviation)[e]. The SES will be calculated and compared graphically and in table format for the 3 training conditions.

2). We will examine the interaction term from a 3x2 repeated measures ANOVA using 2 treatment conditions (placebo/pregabalin) as a within-subject independent variable and 3 training conditions (EPT/DPA/C) as a between-subjects independent variable. A significant interaction term will indicate that the difference between placebo and pregabalin is different across training conditions. Planned paired comparisons of treatment difference between each pair of training conditions will be conducted to identify specific pair differences and address the specific study hypotheses of difference between training conditions. If treatment sequences are not able to be collapsed due to significant differences, order of treatment sequence (A-B/B-A) will be included as an additional between-subjects variable, making the ANOVA a 3x2x2.

Comparison of SES between training conditions will also be made between secondary endpoints.

ANOVA analyses will also be conducted on secondary NRS endpoints (current, worst, and walking pain intensity) with the difference that pairwise comparisons between conditions will only be made if the interaction term of the ANOVA is significant at P <= .1. If the interaction term is not significant to at least the .1 level the pairwise comparisons will not be made.

.

To identify baseline characteristics predicting response to training:

Response to training will be quantified by the change in variables from the psychophysical profile conducted at baseline to the same scores at end of study. This will include CoV and function exponent.

1). Change in each variable will be used as the outcome in a multiple linear regression model (separate models for each variable). Participant characteristics at baseline will be entered into the model using a stepwise method (criteria will be specified in the statistical analysis plan, but most likely .05 to enter and .10 to exit the model) and assessed for significance. Final models will be reported for each variable. Baseline variables to be used as predictors are: age, sex, baseline pain (now, in past 24h, worst pain in past 24h), duration of PDN pain, duration of diabetes, and previous treatment experience (number of previous clinical studies, time in previous clinical studies, and number of previous treatments tried).

---

[e] Cohen's *d* is planned on the initial assumption that any differences between treatment sequences will not confound this measure. If there are differences between treatment sequences such that orders cannot be collapsed a modified equivalent of Cohen's d may be substituted if necessary.

2). Identical multiple linear regression models will be constructed using the same predictor variables but using the psychophysical profile variables from the end of study visit. Using scores at end of study as opposed to change from baseline to end of study will provide an analysis of which baseline variables predict final reporting parameters as opposed to change. For example, some variables may predict change by being correlated with extreme baseline states and it may be useful to differentiate between predictors of final high report reliability and those that predict improvement in report reliability (i.e., those who were always reliable and so had little change and those who started with poor reliability and had room to improve a great deal).

3). Additional analyses will be conducted for subgroups of participants in the EPT and DPA training conditions:

3a). For participants who underwent EPT, regression models will be constructed analogous to those described above for the entire population but with the addition of psychophysical function variables at their final training visit (T4) and change in variables from baseline to T4 as predictors.

3b). For participants in the DPA condition, additional regression models will include as a possible predictor their accuracy, in %, of their discrimination between active treatment and placebo from the TEQ and their average confidence ratings from the TEQ.

3c). No additional models will be constructed for the control condition participants.

Additional psychophysical function variables, possibly including but not limited to function intercept, ICC, and R^2 fit to function curve may be analyzed in similar fashion as exploratory alternative endpoints for training response.

6.5.3. Safety Analyses

The safety analysis population includes participants who receive any training or treatment in the study. This means any participant randomized to a training condition, including control, who returns for a second study visit (V2 or T1) regardless of whether that visit is completed or not. Participants withdrawing before the beginning of their second visit (i.e., during Visit 1 or after Visit 1 but before attending another clinic visit) will not be included in the safety population. The number and percentage of participants with AEs will be displayed by system organ class and preferred term. Summaries in terms of severity and relationship to study drug will also be provided. SAEs will be summarized separately in a similar manner. Subject listings of AEs causing discontinuation of study medication and SAEs will be produced.

Vital Signs: Vital signs analysis will include the mean, standard deviation, minimum, maximum, and quartiles at baseline and at the end of each treatment, and the change from baseline to the end of each treatment.

Clinical Laboratory: Laboratory parameters analysis will include the mean, standard deviation, minimum, maximum, and quartiles at baseline and at the end of each treatment, as well clinically significant shifts in laboratory values during each Treatment Period.

Concomitant Medications: Concomitant medications will be analyzed descriptively.

6.6. Interim Analysis

No interim analyses are planned.

6.7. General Statistical Considerations

All statistical analysis will be performed using either SAS for Windows (version 9.2 or higher) or SPSS for Windows (version 19.0 or higher). Subject data listings and tabular presentation of results will be provided. Presentation of summary statistics for continuous variables will include *N*, mean, median, and standard deviation, as well as the minimum and maximum values. For categorical variables, the number and percentage of each category within a parameter for nonmissing data will be calculated. All clinically relevant baseline variables will be tabulated and compared between the 2 treatment groups and the 3 training condition groups. All statistical tests will be 2-sided unless otherwise stated, employing a significance level of 0.05. No adjustments for multiplicity will be made unless otherwise stated. All comparisons planned in the statistical analysis plan will be completed as specified, and fully reported.

Demographic and other baseline characteristics will be summarized. Medical history will be summarized by body system and by number and percentage of participants reporting the history.

Management of dropouts and missing data will depend on their frequency and the nature of the outcome measure. Analysis of the distribution of prognostic factors between participants with data and those without data will be reviewed for significance to assess selection bias. Adjustments for missing data will be performed only if deemed necessary and will be described completely in the statistical analysis plan.

Outlier values will be evaluated for their validity; all data will be included unless judged to be invalid.

7. ADVERSE EVENTS

7.1. Definition of Adverse Events

An AE is defined as any untoward medical occurrence in a subject. This definition does not imply that there is a relationship between the AE and the drug under investigation.

AEs, as defined above, will be documented in the medical record or source document and on study case report form (CRF).

Asentral, Inc. Institutional Review Board approved – August 22, 2013

The Principal Investigator is responsible for the detection and documentation of events meeting the criteria and definition of an AE or SAE, as provided in this protocol. During the study when there is a safety evaluation, the Investigator or site staff will be responsible for detecting, documenting, and reporting AEs and SAEs, as detailed in this section of the protocol.

Consistent with the current regulatory guidance provided by the US Code of Federal Regulations and the ICH Guideline for Good Clinical Practice, AEs and SAEs are defined below.

Pregabalin is an approved and frequently used prescription treatment for pain from diabetic neuropathy, and AE experience can be found in the package insert.

7.2. Definition of Serious Adverse Events

An AE will be classified as **serious** (an SAE) if it meets any of the following criteria (21 Code of Federal Regulations [CFR] 803.3):

- Results in, or contributes to, a death or serious injury
- Is life-threatening (i.e., the subject was at risk of death at the time of the event)
- Requires subject hospitalization or prolongation of existing hospitalization
- Results in persistent disability or incapacity (i.e., permanent impairment of a body function or permanent damage to a body structure)
- Necessitates medical or surgical intervention to prevent one of the outcomes listed above in this definition (i.e., to preclude permanent impairment of a body function or permanent damage to a body structure)

7.3. Relationship of Adverse Events to Study Medications

The Investigator will use the following categories for assigning the certainty of the relatedness:

- **Definitely Related:** There is a **possible** or probable relationship (i.e., there is a reasonable or strong temporal relationship, and the events are unlikely to be attributable to other drugs, underlying diseases, or other factors).
- **Possibly Related:** An AE is **possibly** related if it is capable of being related but relatively unlikely.
- **Not Related:** The relationship is **unlikely or nonexistent** (i.e., there is no strong temporal relationship and/or the use of other drugs, underlying diseases, or other factors provide plausible explanations for the event), or the subject did not take the investigational product.

If 1 of the above categories cannot be assigned, then use:

- **Unknown:** Use this term if there is insufficient information to determine if the AE is related to the drug.

7.4. Severity of Adverse Events

Each AE should be assessed for its severity, or the intensity of an event, experienced by the subject.

- **Mild:** Awareness of a sign or symptom that does not interfere with the subject's activity or is transient and is resolved without treatment or sequelae.
- **Moderate:** May interfere with the subject's activity and require additional intervention and/or treatment, and may have additional sequelae.
- **Severe:** Significant discomfort to the subject and/or interferes with the subject's activity. Additional intervention and/or treatment are necessary. Additional sequelae occur. "Severe" is used to describe the intensity of an event experienced by the subject.

7.5. Reporting of Adverse Events

If an AE occurs, all sections of the appropriate CRF must be completed, including rating the severity of the event, relationship of the event to study medications, treatments used for the event and outcomes.

All SAEs (including deaths) regardless of cause and whether anticipated or unanticipated must be reported to the Sponsor as soon as possible after the Investigator becomes aware of the event. Copies of all relevant documentation (i.e., dosage reports, physician/nurses notes, discharge summary, etc.) should be submitted to data management and to Sponsor within 72 hours of knowledge of an SAE, or death, as appropriate.

It is the responsibility of the Investigator to inform the IRB of AEs according to IRB requirements. All events reported to the IRB must be documented on the appropriate CRF.

Supplemental information should be submitted as soon as available and may include laboratory results, radiology reports, progress notes, hospital admission and emergency room notes, holding and observation notes, discharge summaries, autopsy reports, and death certificates.

The Sponsor will follow appropriate guidelines for expedited reporting to the U.S. Food and Drug Administration (FDA).

8. CASE REPORT FORMS

The Investigator is responsible for ensuring the accuracy and completeness of all study documentation. All clinical study data will be recorded in the CRFs provided to the investigational site. Completed CRF will be reviewed along with the source documents by the Sponsor or Sponsor designee to ensure accuracy and completeness. The site will ensure

that the medical records are made available for review by the study monitor and any FDA or other government regulatory bodies as required.

The Investigator agrees to maintain accurate source documentation and CRF as part of the case histories.

9. ADMINISTRATIVE REQUIREMENTS

9.1. Investigator Selection

The Investigator must be of good standing as an Investigator and not de-barred by the FDA. The Investigators must be knowledgeable in relevant areas of clinical research to ensure adherence to the requirements of the protocol, including the protection of human participants. Other site personnel must have appropriate research experience and infrastructure to ensure adherence to the protocol and enrollment of sufficient numbers of evaluable participants. The curriculum vitae of the Investigator will be maintained in the Sponsor files as documentation of previous medical training, and federal databases will be searched to ensure that the Investigator and/or the site are not prohibited from engaging in federally sponsored clinical research. The Principal Investigator will sign the signature page of this protocol, agreeing to comply with all applicable government regulations and the requirements of this study.

9.2. Regulatory and Ethical Considerations

Analgesic Solutions will obtain favorable opinion/approval to conduct the study from the appropriate regulatory agency in accordance with any applicable country-specific regulatory requirements prior to a site initiating the study in that country.

9.3. Institutional Review Board Approval

Before commencement of the study, the Investigator must provide Sponsor with written documentation of IRB approval. This approval must refer to the ICF and the study by both the title and the protocol number assigned by Sponsor. The Investigator, if a member of the IRB, is not to participate in the approval decision for this study. This non-participation should be noted in the approval letter. A list of IRB/Ethics Committee voting members, their titles or occupations, their institutional affiliations, or a letter stating that the IRB/Ethics Committee is properly constituted under governing regulations must also be provided to Sponsor or designee. The Investigator is responsible for reporting the following to the IRB/Ethics Committee:

- All SAEs (including deaths), regardless of cause and whether anticipated or unanticipated, to be reported as soon as possible after Investigator becomes aware of the event
- Significant findings that become known in the course of the study and that might affect the willingness of participants to continue to participate

- Protocol, consent, amendments prior to the implementation of the change
- Study progress reports at least once a year, if applicable
- Notification of study completion or termination.

No drug supplies, if applicable, will be shipped to the Investigator until the IRB approval has been supplied to Sponsor and all relevant agreements have been executed.

The IRB must give written renewal of the original approval at least annually to continue the study. A copy of the written renewal must be provided to Sponsor.

9.4. Informed Consent

Prior to the treatment, the Investigator must explain to each subject (or the subject's legally authorized representative) the nature of the study, its purpose, its expected duration, and the benefits and risks of study participation. After this explanation and before entering the study, the subject (or legally authorized representative) must voluntarily sign and date the IRB-approved ICF

9.5. Protocol Adherence and Amendment

The study will be conducted as described in this protocol. The Investigator agrees to conduct this study in accordance with this protocol. Any deviations from this protocol must be documented by the Investigator and reported to the study monitor as soon as possible. If an emergency situation arises in which the safety and welfare of a subject may require immediate alternative intervention, the Investigator should act in the best interests of the subject. Sponsor and the physician's IRB must be notified immediately if this occurs. This should be followed with written confirmation that describes the emergency action and outcomes, to Sponsor and the IRB within 10 working days.

Sponsor or designee may amend the protocol as needed to ensure that the clinical investigation is being conducted as intended. Sponsor will initiate protocol amendments in writing if any change significantly affects the safety of participants, the scope of the investigation, or the scientific quality of the study. Protocol changes must be submitted to the IRB/Ethics Committee as a protocol amendment. If necessary, the ICF will be revised to reflect the changes in the amendment and will be submitted to the IRB/Ethics Committee for review and approval. A copy of the amendment must be signed by the Investigator and returned to Sponsor or designee. Written documentation of IRB/Ethics Committee approval is required before the amendment is implemented.

9.6. Drug Information

9.6.1. General Information on Pregabalin

Pregabalin (Lyrica®) is an FDA-approved gabapentinoid drug for the treatment of pain associated with diabetic neuropathy; it is an anticonvulsant drug that is used for the treatment of pain associated with diabetic neuropathy and as an adjunct therapy for partial

seizures with or without secondary generalization in adults. It is also approved for fibromyalgia, pain after shingles, and partial onset of seizures with epilepsy in people who take 1 or more drugs for seizures.

9.6.2. General information on oxycodone

Oxycodone is an FDA-approved opioid drug for the treatment of moderate to severe pain, commonly used in a wide variety of indications including pain associated with diabetic neuropathy. It is used in both chronic and acute conditions. Oxycodone can cause respiratory depression or hypertension and can be addictive.
Only a single dose of oxycodone will be administered during this study and patients will remain in-clinic for a 4-hour observation period after dosing.

9.6.3. Clinical Supplies

The Sponsor will supply the Investigator with an adequate amount of investigational drug for completion of the study. Clinical supplies are to be stored and maintained at room temperature in secured storage. Reasonable effort will be made to avoid exposure of the drugs to extreme temperatures and humidity.

9.6.4. Drug Accountability

The study drug may only be used for participants enrolled and randomized into this study under the supervision of the Investigator and under the terms of the clinical protocol. The Investigator may not provide the drugs to any person not authorized to use them. The Investigator will maintain drug accountability records. These may include:

- Product code
- Lot number
- Serial number
- Receipt dates
- Dates and quantities dispensed, including subject number and initials if applicable
- Dates and quantities returned by subject number and initials if applicable
- Return date to the Sponsor

At the end of the study, any drugs that have not been used should be returned to the Sponsor as per the instructions provided by the Sponsor.

9.6.5. Potential Risks and Discomforts

From LYRICA Prescribing Information (revision 6/2012) [3]

- Angioedema (e.g., swelling of the throat, head and neck) can occur, and may be associated with life-threatening respiratory compromise requiring emergency treatment. Discontinue LYRICA immediately in these cases.

- Hypersensitivity reactions (e.g., hives, dyspnea, and wheezing) can occur. Discontinue LYRICA immediately in these patients.
- Increased seizure frequency may occur in patients with seizure disorders if LYRICA is rapidly discontinued. Withdraw LYRICA gradually over a minimum of 1 week.[f]
- Antiepileptic drugs, including LYRICA, increase the risk of suicidal thoughts or behavior.
- LYRICA may cause peripheral edema. Exercise caution when coadministering LYRICA and thiazolidinedione antidiabetic agents.
- LYRICA may cause dizziness and somnolence and impair patients' ability to drive or operate machinery.
- Most common adverse reactions (≥ 5% and twice placebo) are dizziness, somnolence, dry mouth, edema, blurred vision, weight gain and thinking abnormal (primarily difficulty with concentration/attention).

Additionally, the effects of pregabalin on human reproduction are unknown. Participants will be excluded from the study if they (for men) plan to father a child or (for women) plan to become pregnant or breast feed.

9.6.6. Breaking of the Blind

9.6.6.1. During Training Evaluation stage

In the event that an emergency unblinding is required, authorized/approved randomization system users, at the study centers and Sponsor (or designee), will have the ability to retrieve subject treatment groups assignment through the randomization system. Unblinding a subject should only be done in emergency situations for reasons of subject safety. The investigator/study center should make every attempt to contact the sponsor's or designee's Medical Monitor before breaking the blind. When the blinding code is broken, the reason must be fully documented. If the blind is broken for any subject, the Investigator must notify the Sponsor as soon as possible.

9.6.6.2. During DPA training

Treatments administered during DPA training sessions are intentionally unblinded after treatment, per protocol, as described above in Section 5.6.2.6. In the event of an emergency requiring unblinding during a DPA training session prior to per-protocol unblinding the Investigator will have the ability to break the blind by referring to the

[f] Note: patients with seizure disorders are excluded from this study.

information that would have been used for unblinding at the end of the training session. If the blind is broken prematurely, this must be documented in the source documentation and the Sponsor notified as soon as possible.

9.7. Data Collection

The Investigator is responsible for completely and accurately recording study data in the appropriate sections of the CRFs provided by Sponsor. The CRFs must be signed by the Investigator or by his/her documented designee.

The monitor will ensure the quality of data recording at each investigational site by comparison to supporting source documents during periodic site visits. Adherence to proper recording of information as well as assuring that corrections are being made will also be addressed during these periodic visits.

9.8. Data Management

Data from CRFs and other external data (e.g., laboratory data) will be entered into a clinical database as specified in the Data Management Plan. Quality control and data validation procedures will be applied to ensure the validity and accuracy of the clinical database.

9.9. Database Quality Assurance

The clinical database will be reviewed and checked for omissions, apparent errors, and values requiring further clarification using computerized and manual procedures. Data queries requiring clarification will be documented and returned to the investigational site for resolution. Only authorized personnel will make corrections to the clinical database, and all corrections will be documented in an audit trail. A quality assurance audit will be performed prior to database lock.

9.10. Required Documentation

An Investigator may not screen or enroll participants until authorized to do so by the Sponsor and IRB approval has been obtained. At a minimum, the following documentation must be received by the Sponsor prior to study commencement:

- Curriculum vitae for the Principal Investigator and Sub-Investigators
- Signed Investigator Agreement
- Signed Confidentiality Statement for Investigator and Sub-Investigators
- Signed "Protocol Agreement Page" (page iii of this protocol)
- Written approval from the IRB/Ethics Committee of both the protocol and informed consent form
- Signed Financial Disclosure Statement
- IRB Assurance of Compliance Form or equivalent

10. SITE MONITORING

The study monitors are designated as agents of the Sponsor and are assigned to oversee the conduct and progress of the study and to be the principal communication link between Sponsor and Investigator. The study monitors will be involved in Investigator selection and training, assurance of IRB/Ethics Committee approvals, and periodic on-site inspection and monitoring of sites and records, to ensure continued compliance with the protocol and adequacy of the Investigator and the facility to carry out the study. In addition, the monitor will verify that the drug is being used in accordance with the protocol instructions. The monitor will perform several types of site visits during the study. Source documents will be reviewed for verification of agreement with data on the CRF. It is important that the Investigator and relevant personnel are available during the monitoring visits and that sufficient time is devoted to the process.

11. TERMINATION OF STUDY

Sponsor reserves the right to suspend enrollment or terminate the study at any time as set forth in the Clinical Study Agreement. Written notice will be submitted to the Investigator in advance of such termination.

Sponsor may suspend enrollment or terminate the study at a specific site for reasons including, but not limited to, inadequate data collection, low subject enrollment rate, achievement of the total enrollment, or noncompliance with the protocol or other clinical research requirements.

12. REPORTING REQUIREMENTS

The Investigator must promptly report to Sponsor any withdrawal of IRB approval at the site. Additional reporting requirements of the Investigator include:

- Reporting all informed-consent violations to the IRB
- Reporting any SAEs to Sponsor

13. RECORD RETENTION

All source documents, CRFs, and ICFs should be retained by the site for no less than 2 years after study completion or termination or until the records are no longer required as determined by Sponsor**.**

The records must be maintained to allow easy and timely retrieval when needed (e.g., audit or inspection), and, whenever feasible, to allow any subsequent review of data in conjunction with assessment of the facility, supporting systems, and staff. Where permitted by local laws/regulations or institutional policy, some or all of these records can be maintained in a format other than hard copy (e.g., microfiche, scanned, electronic); however, caution needs to be exercised before such action is taken. The Investigator must

ensure that all reproductions are legible and are a true and accurate copy of the original and meet accessibility and retrieval standards, including regenerating a hard copy, if required. Furthermore, the Investigator must ensure that there is an acceptable back-up of these reproductions and that an acceptable quality-control process exists for making these reproductions.

Sponsor will inform the Investigator of the time period for retaining these records to comply with all applicable regulatory requirements.

The Investigator must notify Sponsor of any changes in the archival arrangements, including, but not limited to, the following: archival at an off-site facility and transfer of ownership of the records in the event the Investigator leaves the site.

13.1. Financial Disclosure

Consistent with Title 21 CFR Part 54, all Investigators will complete a Financial Disclosure Form that permits Sponsor to demonstrate that an Investigator has no personal or professional financial incentive regarding study outcome or the future approval or disapproval of an investigational drug such that the Investigator's research might be biased by such incentive.

13.2. Use of Information and Disclosure of Data

It is understood by the Investigator that the information obtained through this study will be used by Sponsor in connection with the development of the investigational drug and, therefore, may be used in submission(s) to regulatory authorities. In addition, the results of this study may be used in publications or presented at scientific meetings.

14. REFERENCES

Baumann M, Moffat G, Roberts L, Ward L. Constrained scaling: Achieving quantitative convergence across laboratories. *Fechner Day 2004*. Coimbra, Portugal: International Society for Psychophysics; 2004.

Fedele L, Marchini M, Acaia B, Garagiola U, Tiengo M. Dynamics and significance of placebo response in primary dysmenorrhea. *Pain*. 1989;36:43-47.

Lesser M, Sharma U, LaMoreaux L, Poole R. Pregabalin relieves symptoms of painful diabetic neuropathy: A randomized controlled trial. *Neurology*. 2004;63:2104-2110.

LYRICA® (pregabalin) package insert. Pfizer, Inc. June 2012.

Quiton R, Greenspan J. Across- and within-session variability of ratings of painful contact heat stimuli. *Pain*. 2008:245-256.

Richter R, Portenoy R, Sharma U, Lamoreaux L, Bockbrader H, Knapp L. Relief of painful diabetic peripheral neuropathy with pregabalin: A randomized, placebo-controlled trial. *The Journal of Pain*. 2005;6(4):253-260.

Rosenstock J, Ruchman M, LaMoreaux L, Sharma U. Pregabalin for the treatment of painful diabetic neuropathy: A double-blind, placebo-controlled trial. *Pain*. 2004;110:628-38.

15. APPENDICES

15.1. FAST Assessment

The FAST procedure is a subject selection tool designed by Analgesic Solutions and consists of the following 2 parts: Psychological Assessment and Psychophysical Assessment.

Psychosocial Assessment: The Psychosocial Assessment consists of a series of psychological survey questions that will be presented to the participant, and participant responses will be collected as described in the FAST Instructions Manual. The participant will have as much time to answer each survey question as needed. The Psychosocial Assessment will consist of the surveys as detailed in the FAST Instructions Manual.

Psychophysical Assessment: Participants will be seated comfortably in a chair, and the study procedures will be explained. Participants will be familiarized with the Multimodal Automated Sensory Testing (MAST) system that applies precisely computer-controlled pressure stimuli to the thumbnail (see Appendix 15.7). The MAST device can apply pressure up to10kg/cm2, which can be quite painful but does not cause lasting tissue damage. Participants may experience a temporary tenderness or sensitivity in the thumb, but this typically fades in less than a day. Participants will be told that they may ask questions, express concerns, or stop the procedure at any time by saying “stop,” and the Investigator will stop the procedure.

The participant will place the thumb of their non-dominant hand in the MAST handpiece, with the forearm supported (as by a table or the arm of a chair) and the arm bent comfortably.

The MAST device will apply an ascending series of stimuli to the thumbnail, beginning at .5kg and increasing by .5kg per trial with a pause between trials (interstimulus interval, or ISI) of 20 seconds. Pressure will be applied at a ramp rate of 4kg per second and each stimulus will last approximately 4 seconds at peak pressure. The MAST device automatically self-calibrates the stimulus pressure to maintain a stable force profile across the stimulus. At the conclusion of the stimulus the participant will indicate the intensity of pain they experienced during the stimulus on a 0-10 NRS with 0 being “no pain” and 10 being “Worst Pain Imaginable using the touch-screen client computer of the MAST system. The first pressure at which the participant reports non-zero pain is the pain threshold. Participants are explicitly instructed that ratings should be of pain, not pressure. Trials continue in increasing increments until the participant either gives a rating of maximum pain (10 out of 10) or indicates that they cannot tolerate higher pressure. This pressure is the pain tolerance. The participant may terminate any trial at any time with a simple on-screen button or by verbal communication to the experimenter.

The participant then completes a psychophysical profile procedure in which they rate, on the 0-10 NRS, a series of randomized stimuli between a lower bound of their threshold level and an upper bound of 75% of the range to their tolerance level. The psychophysical profile stimuli are at 6 evenly-spaced intervals between the lower and upper bounds. For example, for a participant with a threshold level of 2kg and tolerance of 6kg would have a lower bound of 2kg and an upper bound of 5kg and intervals would be 2kg, 2.5, 3.0, 3.5, 4.0, and 4.5. Each stimulus level is repeated 4 times for a total of 24 stimuli in randomized order with an ISI of 20 seconds between each trial.

If the subject needs to stop the procedure, he or she may touch the “STOP” button on the MAST client tablet or say “stop” aloud and the Investigator will stop the trial. Continuation of the psychophysical profile procedure will be at the discretion of participant and the Investigator, who will take into consideration the safety and best interests of the participant.

15.2. Evoked Pain Training (EPT)

Evoked pain stimuli will be pressure applied to the participants' thumbnail using the computer-controlled Multimodal Automated Sensory Testing (MAST) system.

Evoked pain training (EPT) consists of 4 stages: 1) assessment of pain threshold and pain tolerance using the MAST, 2) rating of PDN pain using traditional rating scale (e.g., NRS or VAS, whichever is used in the protocol that training is being applied for) and cross-modality matching to pressure pain using MAST, 3) ratings of randomized painful pressure stimuli, and 4) feedback on performance.

Evoked pain stimuli are delivered to the participants' thumbnail via the MAST system. The MAST system is a non-significant risk device which applies a computer-controlled pressure stimulus to the thumbnail at a precisely controlled intensity for a specified duration. The MAST consists of two touchscreen-enabled netbook or laptop computers, one an experimenter control console or server and the other a participant response or client that can display instructions and the participant uses to enter responses, and two handsets that can apply the thumbnail pressure stimulus (only one is used in the EPT procedure). The handset is a pistol-grip style unit with a slot that the participant inserts their thumb into. A rubber-tipped plunger depresses onto the participant's thumbnail with a specified pressure, self-adjusting to the resistance of the thumb and any movement to ensure a consistent pressure. A typical experiment using the MAST would apply a stimulus and then ask the participant to rate that stimulus on a VAS or NRS scale using the client console computer. A detailed description of the MAST safety features and risk assessment are included as Appendix 15.7 of this protocol.

Evoked Pain Training consists of 4 basic stages:

15.2.1. Assessment of Pain Threshold and Tolerance

The MAST device will apply an ascending series of stimuli to the thumbnail, beginning at .5kg and increasing by .5kg per trial with a pause between trials (interstimulus interval, or ISI) of 20 seconds. Pressure will be applied at a ramp rate of 4kg per second and each stimulus will last approximately 4 seconds at peak pressure. The MAST device automatically self-calibrates the stimulus pressure to maintain a stable force profile across the stimulus. At the conclusion of the stimulus the participant will indicate the intensity of pain they experienced during the stimulus on a 0-10 NRS with 0 being "no pain" and 10 being "Worst Pain Imaginable" using the touch-screen client computer of the MAST system. The first pressure at which the participant reports non-zero pain is the pain threshold. Participants are explicitly instructed that ratings should be of <u>pain</u>, not pressure. Trials continue in increasing increments until the participant either gives a rating of maximum pain (10 out of 10) or indicates that they cannot tolerate higher pressure. This pressure is the pain tolerance. The participant may terminate any trial at any time with a simple on-screen button or by verbal communication to the experimenter.

15.2.2. Index Pain Assessment

Participants are asked to provide rating of their current PDN pain 1) using a 0-10 NRS or equivalent VAS and 2) by matching their current pain to evoked pressure pain using an ascending method of limits. The MAST will apply gradually increasing pressure at a rate of .5kg per second to the thumbnail until the participant indicates that the pressure pain is of equal intensity to their PDN pain. This will be done 3 times and the scores averaged.

15.2.3. Random Magnitude Estimation

The primary evoked pain experience used in training is the repeated application and rating of randomly selected pressure stimuli. Stimuli will range from a minimum at the participant's pain threshold and to a maximum of the participant's pain tolerance. The psychophysical profile stimuli are at 6 evenly spaced intervals beginning at the participant's threshold and ending at the last .5kg increment prior to tolerance level. For example, for a participant with a threshold level of 2kg and tolerance of 5kg, intervals would be 2kg, 2.5, 3.0, 3.5, 4.0, and 4.5. Each stimulus level is repeated 4 times for a total of 24 stimuli in randomized order with an ISI of 20 seconds.

15.2.4. Training Feedback

After completing a cycle of magnitude estimations participants will receive feedback on their performance including discussion with the experimenter and review a graphical representation of their responses. An example of a feedback figure is shown in Figure 2: EPT Feedback. Feedback includes, but is not strictly limited to, an explanation of the figure, instructions to attend to and report only the pain sensation, the importance of consistency in reporting, etc.

Figure 2 :EPT Feedback

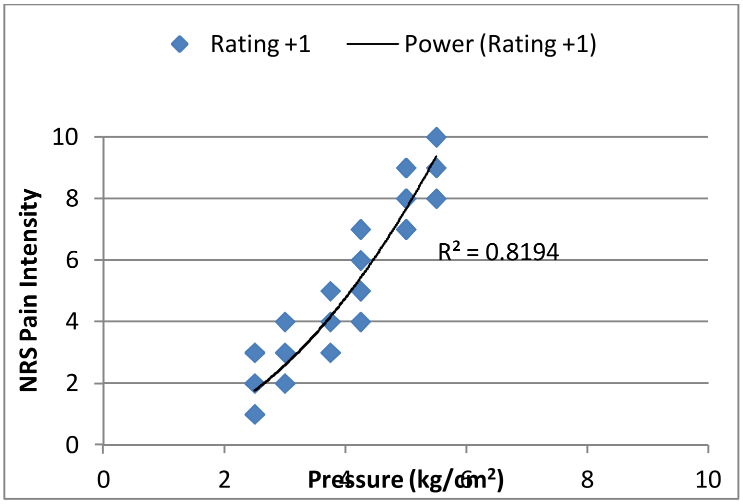

The figure shown is an example; the data shown will be the participant's own.

The 4 steps of EPT are considered one training 'cycle'. There is a break between cycles of at least 5 minutes, but may be longer if the participant desires. The first cycle is applied to the participant's dominant hand and subsequent cycles alternate between the dominant and non-dominant hands.

On a participant's first training visit, during which they are familiarized and become comfortable with the procedure only two cycles will be conducted, one on the dominant hand and one on the non-dominant hand. On subsequent training visits when less time is required for familiarization and practice four cycles will be attempted, two on each hand in alternating order. If a participant is unable to tolerate the third and/or fourth cycles (e.g., due to increased sensitivity) this will not be considered a protocol violation. Participants may discontinue any training cycle at any time. Any participant completing a least 75% (18 out of 24 trials) of two cycles may be considered to have completed a training visit. These may be one cycle on each hand or both on the same hand (e.g., in the case of a participant having an acute injury to one thumb partway through the training period, necessitating only one side be used for a session).

15.3. Treatment Experience Questionnaire (TEQ)

√ (Check) the box that describes the treatment you believe you received (choose one).

- ☐ Active treatment (real drug)
- ☐ Placebo (fake drug)

√ (Check) the box that describes how confident you are of that answer (choose one).

- ☐ Not at all sure
- ☐ Somewhat sure
- ☐ Very sure
- ☐ Positive

Why do you believe you received the active drug or placebo? (Write in any answer)

____

____

____

____

15.4. Pain Intensity NRS

Participants will be asked:

- **Current Pain Intensity NRS:**

On a 0-10 scale, please rate your pain by indicating the number that best describes the intensity of your pain from diabetes **right now**.

0 1 2 3 4 5 6 7 8 9 10

↑ ↑

(No pain) (Worst Pain Imaginable)

- **24-Hour Recall Average Pain Intensity NRS**

On a 0-10 scale, please rate your pain by indicating the number that best describes the **average** intensity of your pain from diabetes **over the past 24 hours**.

0 1 2 3 4 5 6 7 8 9 10

↑ ↑

(No pain) (Worst Pain Imaginable)

- **24-Hour Recall Worst Pain Intensity NRS**

On a 0-10 scale, please rate your pain by indicating the number that best describes the **worst** intensity of your pain from diabetes **over the past 24 hours**.

0 1 2 3 4 5 6 7 8 9 10

↑ ↑

(No pain) (Worst Pain Imaginable)

- **24-Hour Recall Walking Pain Intensity NRS**

On a 0-10 scale, please rate your pain by indicating the number that best describes the **average** intensity of your pain **when walking over the past 24 hours**.

0 1 2 3 4 5 6 7 8 9 10

↑ ↑

(No pain) (Worst Pain Imaginable)

15.5. Patient Global Impression of Change

Participants completing a treatment period will be asked:

How do you feel your pain from diabetes has changed, if at all, after this treatment compared with before the treatment?

Participants will be asked to choose one of the following answers:

Choose only ONE response.

Very Much Better

Much Better

Minimally Better

No Change

Minimally Worse

Much Worse

Very Much Worse

15.6. Patient Preference for Treatment

Treatment Preference Questionnaire – comparison of Treatment Period A versus Treatment Period B

Participants that complete both treatment periods will be asked:

In which treatment period did you receive the medication that relieved your pain most effectively?

Participants will be asked to choose one of the following answers:

Choose only ONE response.

- ☐ First Treatment Much Better than Second Treatment
- ☐ First Treatment Slightly Better than Second Treatment
- ☐ Both Treatments the Same
- ☐ Second Treatment Slightly Better than First Treatment
- ☐ Second Treatment Much Better than First Treatment

15.7. Participant Training Feedback Questionnaire

Participants assigned to EPT and DPA training conditions will complete this feedback form at the end of their last training visit. This includes any participants withdrawing early from the study, but will be at the end of T4 for participants completing a full training sequence.

Participants will be asked:

- **Training Burden**

  On a 0-10 scale, please rate how burdensome the training was for you (how difficult it was to complete or how uncomfortable you found it.

  0 1 2 3 4 5 6 7 8 9 10

  ↑ ↑

  Easy/no discomfort Extremely difficult/uncomfortable

- **Willingness to repeat**

  On a 0-10 scale, please rate how willing you might be to repeat such training in the future (for example in another study).

  0 1 2 3 4 5 6 7 8 9 10

  ↑ ↑

  Definitely would not Definitely would

- **Free response**

  Is there anything that you think of might have made the training more effective or less burdensome for you?

  <free text response>

Asentral, Inc. Institutional Review Board approved – August 22, 2013

15.8. BMI Calculation

Body mass index (BMI) is determined by weight and height according to the following equation: BMI = kg/m2. The BMI for various weights and heights is also referenced at http://nhlbisupport.com/bmi/.

WEIGHT (lbs)

HEIGHT (in)	120	130	140	150	160	170	180	190	200	210	220	230	240	250	260	270	280	290	300	310	320
4'10"	25	27	29	31	34	36	38	40	42	44	46	48	50	52	54	57	59	61	63	65	67
4'11"	24	26	28	30	32	34	36	38	40	43	45	47	49	51	53	55	57	59	61	63	65
5'0"	23	25	27	29	31	33	35	37	39	41	43	45	47	49	51	53	55	57	59	61	63
5'1"	23	25	27	28	30	32	34	36	38	40	42	44	45	47	49	51	53	55	57	59	61
5'2"	22	24	26	27	29	31	33	35	37	38	40	42	44	46	48	49	51	53	55	57	59
5'3"	21	23	25	27	28	30	32	34	36	37	39	41	43	44	46	48	50	51	53	55	57
5'4"	21	22	24	26	28	29	31	33	34	36	38	40	41	43	45	46	48	50	52	53	55
5'5"	20	22	23	25	27	28	30	32	33	35	37	38	40	42	43	45	47	48	50	52	53
5'6"	19	21	23	24	26	27	29	31	32	34	36	37	39	40	42	44	45	47	49	50	52
5'7"	19	20	22	24	25	27	28	30	31	33	35	36	38	39	41	42	44	46	47	49	50
5'8"	18	20	21	23	24	26	27	29	30	32	34	35	37	38	40	41	43	44	46	47	49
5'9"	18	19	21	22	24	25	27	28	30	31	33	34	36	37	38	40	41	43	44	46	47
5'10"	17	19	20	22	23	24	26	27	29	30	32	33	35	36	37	39	40	42	43	45	46
5'11"	17	18	20	21	22	24	25	27	28	29	31	32	34	35	36	38	39	41	42	43	45
6'0"	16	18	19	20	22	23	24	26	27	29	30	31	33	34	35	37	38	39	41	42	43
6'1"	16	17	19	20	21	22	24	25	26	28	29	30	32	33	34	36	37	38	40	41	42
6'2"	15	17	18	19	21	22	23	24	26	27	28	30	31	32	33	35	36	37	39	40	41

Reference: Center for Disease Control and Prevention. (2004, April). Body Mass Index Formula. Retrieved July 16, 2005 from http://www.cdc.gov/nccdphp/dnpa/bmi.

15.9. MAST System Safety Features and Risk Determination

15.9.1. MAST System Safety Features

The MAST handpiece employs several methods to avoid patient injury and maintain safety. These methods are categorized in three components of the system hierarchy, specifically mechanical, electrical and software.

Mechanical Safety Features

The most fundamental safety features rely on the mechanical design of the device. Passive methods include the large, easily accessible mechanical power switch that is able to instantly remove all power to the device (including the motor). In conjunction with this, an aluminum shaft is connected to the pinion that drives the plunger. This shaft can be used to manually move the plunger and remove the force applied to the patient in the event of an electrical or control system failure. The motor and gear system has been selected so that it is physically unable to provide more than approximately 200N (approximately 20kg/cm^2) of force to the thumb, preventing severe and/or permanent tissue or bone damage.

Electrical System Safety Features

On the electrical side, fuses have been included on the output of the battery to prevent excessive current flowing in the control circuitry and producing excessive heating. An absolute rotary encoder has been attached to the motor output shaft so that the position of the plunger is continuously monitored. If the plunger moves out of the hard-coded operating range, the power to the motor is immediately removed. This range has been set to ensure the motor will not drive the plunger all the way to the bottom of the testing area. In addition, a load cell has been integrated into the plunger and measures the force directly applied 50 times per second. If the command force is exceeded by 25N, the testing is immediately terminated and the plunger retracted. There are also additional internal limits that can be set to limit the maximum power delivered to the motor and hence the maximum force that can be applied. Due to the nature of these limits, they are inherently less accurate than the load cell, but they provide additional safety in the unlikely event of the load cell under-reporting the applied force and the controller continually applying more power to try to achieve the set point.

Software Safety Features

The device has also been designed to independently control each stimulus interval. This means that the controlling computer will set up the required parameters for the stimulus (i.e., force and duration) and give a “go” command that will be transmitted to the handpiece via a Bluetooth link. The device will then have full control of the stimulus application until it is complete. This is done to prevent lost or corrupted communications interfering with the force profile applied to the patient. Besides the inherent error detection built-in to the Bluetooth protocol, the device will echo any received commands back to the controlling computer, so that the validity of test parameters can be verified prior to starting a stimulus. However, Bluetooth operates in the unlicensed 2.4 GHz ISM

frequency range and a Frequency Hopping (FH) algorithm is used to ensure the link is robust to interference. In the event of a situation requiring the test be stopped, the computer software can send a terminate stimulus command that will immediately cause the plunger to retract.

15.9.2. MAST Risk Determination

The MAST System does not require IDE approval because it does not meet the requirements of a Significant Risk (SR) device as defined by the FDA.

The MAST system does not fulfill the definition of a SR device for the following specific reasons:

a) All testing conducted with the MAST System is non-invasive. The device is intended to apply controlled amounts of pressure to peripheral tissue (i.e., the thumbnail bed) only. The testing does not require invasive sampling nor is it intended to introduce energy into a subject.

b) The MAST System is designed exclusively for the assessment of pressure pain sensitivity and global sensory processing in clinical research applications. It is not intended to nor can it support or sustain human life in anyway.

c) The MAST System is not intended for use as a diagnostic device and will be used solely for research applications. Testing will be conducted on consented healthy persons and/or patients that have been diagnosed *a priori* to study enrollment by licensed medical professionals using the accepted tests, devices, and procedures appropriate to the disease under investigation. Furthermore, the MAST System will not be used to cure, mitigate, treat, or prevent disease or other impairments of human health.

d) This device does not pose a serious risk to the health, safety, or welfare of research subjects. The MAST System is used to assess pressure pain sensitivity at the thumbnail in different cohorts of chronic pain patients and healthy controls at multiple time points. This information is used to assess underlying pain mechanisms, the correlation between clinical and evoked pain, longitudinal changes in pain sensitivity, and/or the effectiveness of pain relieving interventions (e.g., drug treatments, surgery, exercise, etc.). Experience by the device developers and others over the last 10 years using similar methods and devices to apply pressure to the thumbnail bed indicate that this procedure is safe and well-tolerated in hundreds of subjects that have been tested over that time. A small number of incidences of thumbnail bruising were reported. Other forms of sustained tissue injury have not been observed. However, subjects are expected to experience pain to the thumb as an intended consequence of the testing. This is a normal response. For most subjects, this pain will dissipate immediately following the removal of the piston from the thumbnail. Some subjects may experience mild thumb tenderness for 1-2 days following testing. In addition, redundant mechanical, electrical, and software safety features have been incorporated into this device to prevent subjects from suffering serious and/or permanent injury. Subjects undergo extensive familiarization with the device, its operation, and its safety features prior

to testing. Moreover, subjects are always permitted to stop testing at any time if the pain becomes intolerable.

If the investigator observes signs of orthostatic reaction from the subject during the administration of the procedure, the procedure will be stopped and the subject will be discontinued from the study.

According to FDA regulations, a non-significant risk (NSR) device is one that does not meet the definition for a SR device (see below). Given that the MAST System does not meet the SR device criteria, we propose that this protocol represents a NSR study.

Under 21 CFR 812.3 (m), an SR device is an investigational device that:

a) Is intended as an implant and presents a potential for serious risk to the health, safety, or welfare of a subject;

b) Is purported or represented to be for use supporting or sustaining human life and presents a potential for serious risk to the health, safety, or welfare of a subject;

c) Is for a use of substantial importance in diagnosing, curing, mitigating, or treating disease, or otherwise preventing impairment of human health and presents a potential for serious risk to the health, safety, or welfare of a subject; or

d) Otherwise presents a potential for serious risk to the health, safety, or welfare of a subject.

Asentral, Inc. Institutional Review Board approved – August 22, 2013
